# In Vitro and In Vivo Analysis of Extracellular Vesicle‐Mediated Metastasis Using a Bright, Red‐Shifted Bioluminescent Reporter Protein

**DOI:** 10.1002/ggn2.202100055

**Published:** 2022-01-19

**Authors:** Gloria I. Perez, David Broadbent, Ahmed A. Zarea, Benedikt Dolgikh, Matthew P. Bernard, Alicia Withrow, Amelia McGill, Victoria Toomajian, Lukose K. Thampy, Jack Harkema, Joel R. Walker, Thomas A. Kirkland, Michael H. Bachmann, Jens Schmidt, Masamitsu Kanada

**Affiliations:** ^1^ Institute for Quantitative Health Science and Engineering (IQ) Michigan State University East Lansing Michigan 48824 USA; ^2^ College of Osteopathic Medicine Michigan State University East Lansing MI 48824 USA; ^3^ College of Natural Science Michigan State University East Lansing MI 48824 USA; ^4^ Department of Pharmacology and Toxicology Michigan State University East Lansing MI 48824 USA; ^5^ Center for Advanced Microscopy Michigan State University East Lansing MI 48824 USA; ^6^ Department of Biomedical Engineering Michigan State University East Lansing MI 48824 USA; ^7^ Promega Biosciences LLC 227 Granada Dr San Luis Obispo CA 93401 USA; ^8^ Department of Microbiology and Molecular Genetics Michigan State University East Lansing MI 48824 USA; ^9^ Department of Obstetrics and Gynecology College of Human Medicine Michigan State University East Lansing MI 48824 USA; ^10^ Department of Biological Sciences Purdue University West Lafayette IN 47906 USA

**Keywords:** autophagy, biodistribution, bioluminescence resonance energy transfer, exosomes, extracellular vesicles, m/lEVs, metastasis, microvesicles, sEVs

## Abstract

Cancer cells produce heterogeneous extracellular vesicles (EVs) as mediators of intercellular communication. This study focuses on a novel method to image EV subtypes and their biodistribution in vivo. A red‐shifted bioluminescence resonance energy transfer (BRET) EV reporter is developed, called PalmReNL, which allows for highly sensitive EV tracking in vitro and in vivo. PalmReNL enables the authors to study the common surface molecules across EV subtypes that determine EV organotropism and their functional differences in cancer progression. Regardless of injection routes, whether retro‐orbital or intraperitoneal, PalmReNL positive EVs, isolated from murine mammary carcinoma cells, localized to the lungs. The early appearance of metastatic foci in the lungs of mammary tumor‐bearing mice following multiple intraperitoneal injections of the medium and large EV (m/lEV)‐enriched fraction derived from mammary carcinoma cells is demonstrated. In addition, the results presented here show that tumor cell‐derived m/lEVs act on distant tissues through upregulating LC3 expression within the lung.

## Graphical abstract

Cancer cell‐derived extracellular vesicles (EVs) promote tumor growth and spread. Studying the distribution of EVs in the body is key to understanding cancer progression and developing therapies. Here, a novel EV reporter for tracking medium and large EVs (m/lEVs) in mice in vivo is developed. Moreover, it is demonstrated that m/lEVs accelerate lung metastasis in mice bearing mammary tumors.

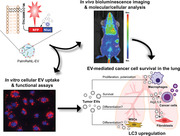

## 1 Introduction

Extracellular vesicles (EVs) are spherical lipid bilayered structures naturally shed by cells and have been implicated in the pathogenesis of cancer and numerous other diseases.^[^ ^1^ ^]^ Understanding their biodistribution and ultimate targets is key to elucidating their roles in health and disease.^[^ ^2^ ^]^ EV subtypes include exosomes and microvesicles (MVs), which are distinguished based on their size and biogenesis.^[^ ^1^ ^]^ Exosomes range from ≈30–120 nm in diameter and are produced by inward budding of the late endosomal membrane, known as multivesicular bodies (MVBs). MVs are 50–1000 nm in diameter and produced by simple outward budding of the plasma membrane. Due to their nanosize and biophysical properties, both types of EVs have the potential to cross biological barriers and gain access into host cells beyond these barriers.^[^ ^3^ ^]^ In this manner, released EVs act as mediators of intercellular communication in the body.^[^ ^4^ ^]^ For example, numerous studies have demonstrated that cancer cells can appropriate this communication pathway by transferring active biomolecules to adjacent and distant cancer cells, promoting their growth and survival.^[^ ^1^, ^5^ ^]^ For this reason, EV‐mediated signaling may hold promising cancer treatment strategies and be an effective platform for drug delivery. However, systemic administration of nanosized EVs may reach and accumulate in other sites beyond the tissues of therapeutic interest.^[^ ^6^ ^]^ Therefore, analysis of EV biodistribution is a prerequisite for the development of EV‐based therapeutics.

Characterization of EV biodistribution is restricted by the biological tools available, which are not sensitive enough to localize and track small EVs in vivo. Commonly used lipophilic fluorescent dyes^[^ ^7^ ^]^ are convenient but lack specificity. These dyes remain intact during EV processing and label recipient cells and tissue over time, causing inaccurate spatiotemporal properties of EVs.^[^ ^8^ ^]^ Alternatively, protein‐based fluorescent or bioluminescent EV reporters (fluorescent proteins,^[^ ^8^, ^9^ ^]^ *Gaussia* luciferase [Gluc],^[^ ^2^, ^10^ ^]^ and NanoLuc^[^ ^6^, ^11^ ^]^) have been developed. Moreover, a recent study demonstrated that PalmGRET,^[^ ^6c^ ^]^ a bioluminescence resonance energy transfer (BRET) EV reporter, enabled multiresolution imaging of extracellular particles (EPs), including exomeres^[^ ^12^ ^]^ (<50 nm) and small EVs (<200 nm; sEVs), medium and large EVs (>200 nm; m/lEVs) categorized according to the Minimal Information for Studies of Extracellular Vesicles (MISEV) 2018 guidelines.^[^ ^13^ ^]^ PalmGRET was created by genetically fusing a palmitoylation signal sequence^[^ ^8a^ ^]^ to a GFP‐NanoLuc BRET reporter (GpNluc^[^ ^14^ ^]^). Wu et al. visualized the lung tropism of PalmGRET‐carrying EPs released from hepatocellular carcinoma (HCC) cells in immunocompetent mice.^[^ ^6c^ ^]^ Notably, the knockdown of some membrane proteins demonstrated distinct biodistribution profiles with decreased lung tropism of HCC cell‐derived reporter EPs.

In this study, we develop a novel red‐shifted BRET EV reporter, PalmReNL, that greatly improves the sensitivity of EV tracking in vivo since bioluminescence imaging produces negligible background signals. Also, photons with spectral wavelengths longer than 600 nm can efficiently penetrate tissues with less light attenuation than observed with shorter wavelength light. Moreover, PalmReNL can be used as a fluorescent EV reporter for standard flow cytometry and microscopy. Non‐invasive in vivo bioluminescence imaging combined with molecular and cellular analyses offers unique potential to facilitate the preclinical evaluation of biological therapies in animal models. Additionally, many in vivo studies of EV‐mediated signaling have only used immune‐compromised mice^[^ ^2^, ^15^ ^]^ and require further assessment in the presence of an intact immune system. In our current studies, combining in vitro and in vivo approaches with immunocompetent mice, we expect to uncover some of the biological characteristics of EVs, such as trafficking, cellular uptake, and release.

## 2 Results

### 2.1 Overexpression of PalmReNL Labeled Both sEVs and m/lEVs

**Figure 1:**
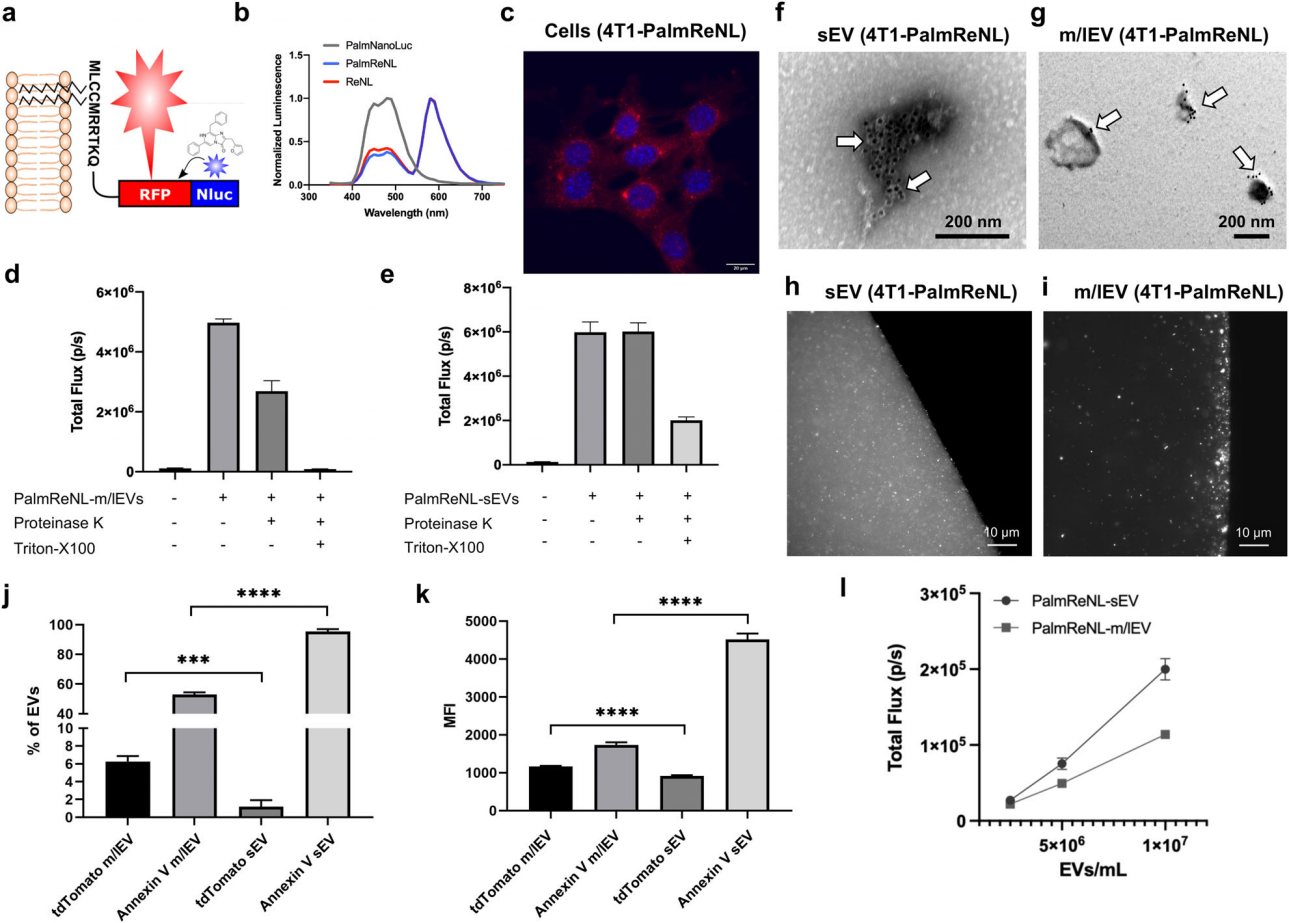
PalmReNL‐based labeling of sEVs and m/lEVs derived from 4T1 cells. a) Schematic diagram of EV membrane labeling with PalmReNL BRET probe. b) Emission spectra of murine mammary carcinoma 4T1 cells transfected with PalmNanoLuc, PalmReNL, or ReNL. Bioluminescence spectra of PalmNanoLuc, PalmReNL, and ReNL were normalized at the 460, 590, and 590 nm luminescence intensities, respectively. c) 4T1 cells stably expressing PalmReNL. Punctate signals of RFP (red; tdTomato) were merged with nuclei stained with Hoechst 33 342 (blue). Scale bar, 20 µm. d,e) Proteinase‐K protection assay for PalmReNL‐m/lEVs and ‐sEVs. f,g) Transmission electron microscopy of 4T1 cell‐derived PalmReNL‐sEVs or ‐m/lEVs, immunogold labeled for tdTomato. Arrows point towards positive tdTomato signals (dark spots). Scale bars, 200 nm. h,i) A droplet of buffer containing isolated PalmReNL‐sEVs and ‐m/lEVs. Scale bars, 10 µm. j,k) The percentage and median fluorescence intensity (MFI) of Annexin V staining of individual PalmReNL‐m/lEVs and ‐sEVs analyzed by flow cytometry. Error bars, SD (*n* = 3), ***p < 0.001; ****p < 0.0001. l) Bioluminescence analysis of PalmReNL‐sEVs and ‐m/lEVs using furimazine. Error bars, SD (*n* = 5).

We developed PalmReNL by genetically fusing a palmitoylation signal sequence^[^ ^8a^ ^]^ to one of the brightest red‐shifted BRET reporters, Red‐eNanoLantern (ReNL)^[^ ^16^ ^]^ (**Figure** **1**a). In our experiments, PalmReNL supplied with its substrate furimazine (Fz) produced red‐shifted luminescence similar to that of ReNL without palmitoylation, indicating that membrane‐anchoring ReNL does not affect its BRET efficiency (Figure 1b). We generated 4T1 cells stably expressing PalmReNL using Sleeping Beauty transposons^[^ ^17^ ^]^ and selected the PalmReNL‐EV donor cells that maintained a high level of PalmReNL expression by limiting dilution (Figure 1c).

As reported previously,^[^ ^8a^ ^]^ we determined that PalmReNL can label the inner membrane leaflet of both sEVs and m/lEVs. However, there were differences in the outer membrane labeling by PalmReNL between sEVs and m/lEVs. As demonstrated by both the dot blot (Figure S1a–d, Supporting Information) and the proteinase‐K protection assays (Figure 1d,e), the reporter was protected from Proteinase‐K in sEVs, hence PalmReNL localizes primarily to the inner membrane. In contrast, PalmReNL was sensitive to Proteinase‐K without detergent treatment in m/lEVs (Figure 1d). This result suggests that PalmReNL localizes to the inner and outer membranes in m/lEVs; however, because of the impurity of the m/lEV‐enriched fraction, we cannot rule out that co‐isolation of EV‐unbound PalmReNL protein aggregates with m/lEVs by centrifugation leads to this Proteinase‐K sensitivity in the absence of detergent. Also, there were signals for sEVs treated with Proteinase‐K and Triton‐X100 (Figure 1e), suggesting some sEV populations may be resistant to detergent treatment. This dual‐labeling of sEVs and m/lEVs represents an advantage over other widely used CD63‐based EV reporters that only label one specific EV subtype in sEVs.^[^ ^18^ ^]^

We next assessed the labeling efficiency in isolated sEV‐ and m/lEV‐enriched fractions from 4T1 cells stably expressing PalmReNL. First, sEVs and m/lEVs were isolated from the conditioned medium as we previously reported^[^ ^8^, ^19^ ^]^ and characterized by nanoparticle tracking analysis (NTA). The concentration of m/lEVs carrying PalmReNL was 2.6 × 10^10^ EVs mL^−1^ and the mean diameter was 121.7 nm (Figure S1f, Supporting Information), while the concentration of sEVs carrying PalmReNL was 1.9 × 10^10^ particles mL^−1^ and the mean diameter was 105 nm (Figure S1e, Supporting Information). The concentrations and mean diameters of sEV‐ and m/lEV‐enriched fractions derived from unmodified 4T1 cells were 1.9 × 10^10^ particles mL^−1^ and 102.4 nm, 2.7 × 10^10^ particles mL^−1^ and 117.2 nm, respectively (Figure S1g,h, Supporting Information). Therefore, the genetic addition of the reporter did not inhibit the release of EVs nor influence their sizes. Of note, whereas the m/lEV fraction contains larger EVs compared to the sEV fraction, their size distributions overlap significantly as previously reported,^[^ ^6^, ^8^ ^]^ indicating that m/lEVs enriched by centrifugation at 20 000 × *g* may contain small (<200 nm in diameter) but higher density EV populations. Determination of the Zeta potential revealed that the PalmReNL slightly shifted the surface charge of sEVs, but not that of m/lEVs (Figure S1i, Supporting Information). Consistent with the NTA data, the transmission electron microscopy (TEM) analysis of the sEV‐ and m/lEV‐enriched fractions revealed a heterogeneous mixture of predominantly intact vesicles with artifactual cup‐shaped morphology^[^ ^20^ ^]^ expressing the tdTomato as ReNL is a fusion protein of NanoLuc and tdTomato^[^ ^16^ ^]^ (Figure 1f,g). There was no significant morphological change in the EV fractions expressing PalmReNL. Notably, we often found PalmReNL proteins in smaller particles (<50 nm in diameter) in the sEV fraction (Figure 1f), but not in typical sEVs (≈100 nm in diameter), possibly due to the limited antibody access to PalmReNL localized to the inner membrane leaflet in sEVs or the inefficient labeling of typical sEVs with PalmReNL.

Western blot (WB) analysis of exosome marker proteins in immunoblots of whole‐cell lysates and EVs derived from unmodified 4T1 cells (Figure S1j, Supporting Information) demonstrated that sEV fractions preferentially express CD63, TSG101, and Alix, whereas m/lEV‐enriched fractions preferentially express Flotillin‐1 as we previously reported.^[^ ^19^ ^]^ Importantly, WB analysis of EVs collected from 4T1 cells stably expressing PalmReNL demonstrated that the reporter protein (PalmReNL) does not interfere with the expression of any of the EV marker proteins tested (Figure S1k, Supporting Information). Both reporter EV fractions contained tdTomato proteins. The labeling efficiency was also confirmed by fluorescence microscopy, demonstrating that the total fluorescence intensities were higher in sEVs, but punctate signal intensity was higher for individual m/lEVs (Figure 1h,i). To further assess the efficiency of EV labeling with PalmReNL, we analyzed individual PalmReNL‐sEVs and ‐m/lEVs by flow cytometry (Figure 1j,k and Figure S1l,m, Supporting Information). First, all the isolated PalmReNL‐EVs were stained with CellTrace Violet (CTV) as an alternative to Carboxyfluorescein succinimidyl ester (CFSE), an amine‐reactive dye previously used for nanoFACS.^[^ ^21^ ^]^ The PalmReNL signal was detected on the tdTomato channel, and the percentage of positive labeling was 1.2% for sEVs and 6.3% for m/lEVs stained with CTV (Figure 1j). The median fluorescence intensity (MFI) of PalmReNL in individual m/lEVs was 1.27‐fold higher than that of sEVs (Figure 1k). As we previously reported, phosphatidylserine (PS) externalization in sEVs and m/lEVs was examined using Annexin V staining.^[^ ^8b^ ^]^ The fluorescence signals in individual sEVs were significantly higher than the signals in individual m/lEVs, where 95.4% and 52.9% of PS externalization was detected in sEVs and m/lEVs stained with CTV, respectively (Figure 1j). The MFI of Annexin V in individual m/lEVs was 2.6‐fold lower than that of sEVs among the CTV‐stained EVs (Figure 1k).

Measuring the bioluminescence signals in equal numbers of PalmReNL‐sEVs and ‐m/lEVs ranging from 2.5 × 10^6^ to 1.0 × 10^7^ EVs mL^−1^ using 25 µm Fz demonstrated that the bioluminescence signals in the sEVs (1.9 × 10^5^ ± 1.4 × 10^4^ p s^−1^; *p *< 0.0001) were 1.7‐fold higher than those of the m/lEVs (1.1 × 10^5^ ± 1.8 × 10^3^ p s^−1^) (Figure 1l). The protein concentrations of 5.6 × 10^9^ EVs/mL were 45.4 µg mL^−1^ for sEVs and 23.9 µg mL^−1^ for m/lEVs. Because of the different sensitivities between bioluminescence and fluorescence, these results suggest that more sEVs may incorporate PalmReNL compared to m/lEVs. Importantly, membrane‐anchoring PalmReNL does not affect its BRET efficiency (Figure 1b), indicating both tdTomato and NanoLuc are fully functional. Therefore, fluorescence signals in some PalmReNL‐sEVs may be below the background autofluorescence, and more m/lEVs carry detectable amounts of PalmReNL molecules due to the larger surface areas relative to sEVs and possibly symmetrical labeling of m/lEV membranes.

### 2.2 Rates of Endocytosis of Tumor Cell‐Derived sEVs and m/lEVs were Similar between Various Recipient Cell Types

**Figure 2:**
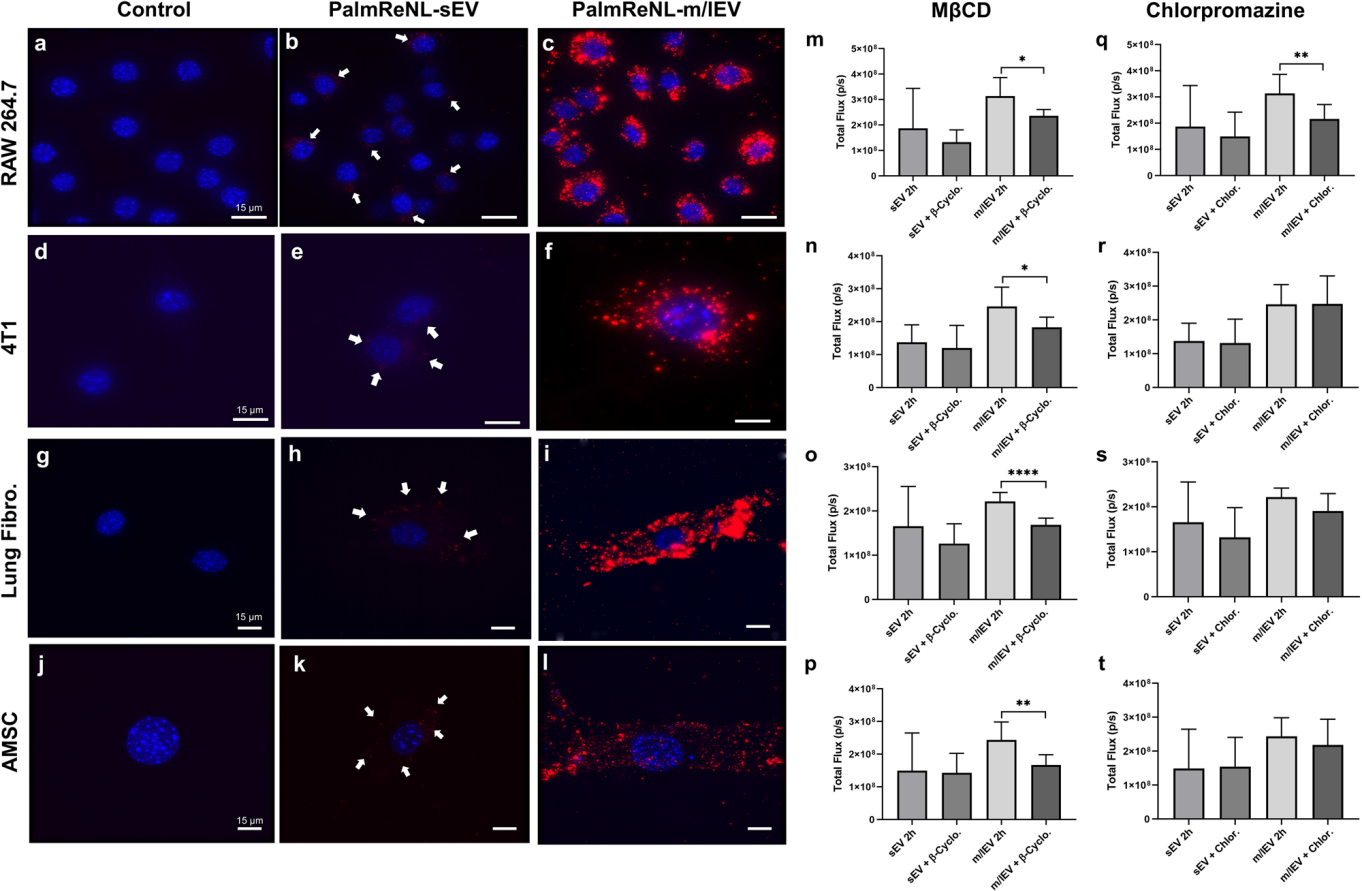
Similar rates of endocytosis of 4T1 cell‐derived PalmReNL‐sEVs and ‐m/lEVs between various recipient cell types in vitro. a–c) Macrophages (RAW 264.7). d–f) 4T1 cells. g–i) primary mouse lung fibroblasts. j–l) Adipose‐derived mesenchymal stromal cells (AMSCs). Punctate signals of tdTomato (red) were merged with nuclei stained with Hoechst 33 342 (blue). Scale bar, 15 µm. Arrows indicate weak RFP signals in PalmReNL‐sEVs. m–t) The recipient cells were treated with methyl‐*β*‐cyclodextrin (MβCD; 10 mm) (m–p) or Chlorpromazine (10 µg mL^−1^) (q–t). m,q) RAW 264.7 cells. n,r) 4T1 cells. o,s) Primary mouse lung fibroblasts. p,t) AMSCs. Error bars, SD (*n* = 8), *p < 0.05; **p < 0.01; ****p < 0.0001.

Uptake of PalmReNL‐sEVs and m/lEVs by macrophages (**Figure** **2**a–c), 4T1 cells (Figure 2d–f), lung fibroblasts (Figure 2g–i), or adipose‐derived mesenchymal stromal cells (AMSCs) were assessed (Figure 2j–l). Phase contrast and fluorescence microscopy demonstrated that EV‐uptake was time‐dependent. In our experimental conditions, the 24 h time point showed the highest fluorescence.

The bioluminescence signal did not reveal any significant differences between the cell types in the uptake of the reporter, except cellular uptake of PalmReNL‐m/lEVs by RAW 264.7 cells and lung fibroblasts. Both cell types had a significantly lower (*p *< 0.05) bioluminescence signal of PalmReNL‐m/lEVs at 24 h (Figure S2a–d, Supporting Information). By contrast, the uptake of PalmReNL‐sEVs appeared to remain constant during the 24 h period in all cell types tested. Next, we assessed the mechanism of cellular uptake of sEVs and m/lEVs. Inhibition of caveolin‐dependent endocytosis, which was inhibited by methyl‐*β*‐cyclodextrin (MβCD; a compound that sequesters the cholesterol in the cell membrane^[^ ^22^ ^]^), significantly decreased the m/lEV‐uptake in all cell types studied (Figure 2m–p). Interestingly, m/lEV uptake in RAW 264.7 cells appears to occur by both clathrin‐dependent endocytosis, which was inhibited by chlorpromazine^[^ ^23^ ^]^ and caveolin‐dependent endocytosis (Figure 2m,q). On the other hand, the uptake of sEVs in all the cell types tested was independent of both caveolin and clathrin (Figure 2m–t).

**Figure 3:**
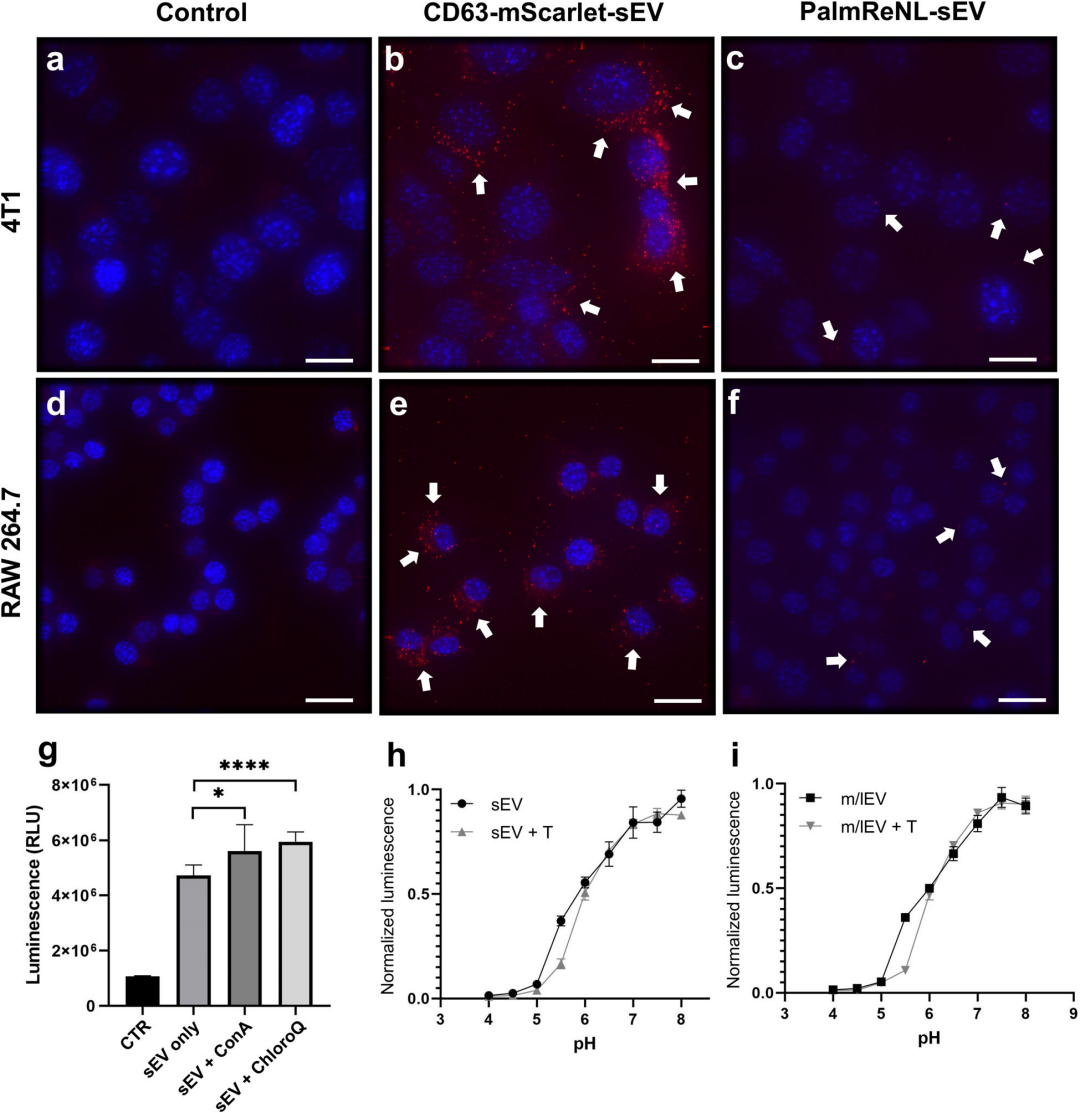
Rapid processing of PalmReNL carried by sEVs via the endosomal‐lysosomal pathway. Fluorescence microscopy images of 4T1 and RAW 264.7 cells treated for 24 h with PalmReNL‐ or CD63‐mScarlet‐sEVs. a,d) Control 4T1 and RAW 264.7 cells. b,e) 4T1 and RAW 264.7 cells treated with CD63‐mScarlet‐sEVs. c,f) 4T1 and RAW 264.7 cells treated with PalmReNL‐sEVs. Punctate fluorescence signals (red) were merged with nuclei stained with Hoechst 33 342 (blue). Scale bar, 15 µm. White arrows, PalmReNL‐ or CD63‐mScarlet‐sEVs. g) PalmReNL‐sEVs taken up by 4T1 cells after 24 h in the presence of concanamycin‐A (0.5 nM) or chloroquine (50 µm) showed higher bioluminescence signals compared to the PalmReNL‐sEV‐treated control. Error bars, SD (*n* = 8), *p < 0.03, ****p < 0.0001. h,i) Conventional pH titration curves of the normalized bioluminescence signals of PalmReNL‐sEVs and ‐m/lEVs with or without Triton X‐100 treatment. Error bars, SD (*n* = 5).

Although the bioluminescence signal for PalmReNL‐sEVs was higher than that of PalmReNL‐m/lEVs (Figure 1l), detecting sEV uptake by fluorescence microscopy was challenging. We hypothesized that our inability to detect the PalmReNL‐sEVs reflects the fact that reporter sEVs cannot retain the fluorescence signal after being taken up by the cells. The rapid diffusion of PalmReNL proteins in endosomal membranes might make their fluorescence signals below the background autofluorescence. To test this, we compared the in vitro uptake by RAW 264.7 macrophages or 4T1 cells of sEVs derived from 4T1 cells stably expressing the exosome marker CD63 fused with mScarlet^[^ ^24^ ^]^ or PalmReNL. CD63‐mScarlet‐sEVs showed punctate fluorescence signals in both recipient 4T1 and RAW 264.7 cells (**Figure** **3**b,e), demonstrating their cellular uptake and signal retention after being taken up by cells. On the other hand, PalmReNL‐sEVs did not retain the fluorescence signals after being taken up by cells and therefore precluded their visualization with the current sensitivity of our microscope (Figure 3c,f). However, PalmReNL‐sEVs retained bioluminescence in the recipient cells (Figure S2, Supporting Information). These results suggest that PalmReNL carried by sEVs might be rapidly transferred from early endosomes into other membrane compartments for either degradation or recycling. On the other hand, transferred CD63‐mScarlet may be retained in endosomal membranes in recipient cells, as recently reported.^[^ ^18^ ^]^ However, we cannot rule out that Palmitoylation and CD63 possibly label distinct sEV populations. In addition, distinct susceptibility to the acidic environment and protein degradation between mScarlet and tdTomato may lead to the difference in fluorescence signal retention.

To further assess the effect of acidic cellular compartments on PalmReNL‐sEVs, we used Palm‐fused Gamillus (acid‐tolerant monomeric GFP^[^ ^25^ ^]^) for EV labeling and compared the uptake of sEVs by fluorescence microscopy. At 24 h, the signal of PalmGamillus‐sEVs was easily detected by fluorescence microscopy compared to that of PalmReNL‐sEVs, which was barely detectable (Figure S3a–d, Supporting Information). Moreover, the treatment of cells with endosomal acidification inhibitors, either Concanamycin A^[^ ^26^ ^]^ or Chloroquine,^[^ ^27^ ^]^ significantly increased (*p *< 0.05) the bioluminescence signal of PalmReNL‐sEVs (Figure 3g). Since the loss of NanoLuc activity with endosomal translocation was previously reported,^[^ ^28^ ^]^ we analyzed the pH sensitivity of PalmReNL‐EVs. The bioluminescence signals of both PalmReNL‐sEVs and ‐m/lEVs steadily decreased at pH below 6.0 either with or without detergent treatment (Figure 3h,i), indicating significant bioluminescence signal loss of PalmReNL‐EVs in acidic cellular compartments. In addition, we assessed the bioluminescence signal retention after cellular uptake of PalmReNL‐EVs. 4T1 cells were cultured with PalmReNL‐sEVs or ‐m/lEVs for 2 h, and the free PalmReNL‐EVs in the media were washed off. The recipient cells were analyzed by measuring bioluminescence signals at various time points. Bioluminescence signals decreased by 71% and 62% at 4 h, and 87% and 91% at 24 h in the recipient cells treated with PalmReNL‐sEVs and ‐m/lEVs, respectively (Figure S3e, Supporting Information).

### 2.3 Proliferation of Various Cell Types Treated with Tumor Cell‐Derived sEVs and m/lEVs In Vitro

**Figure 4:**
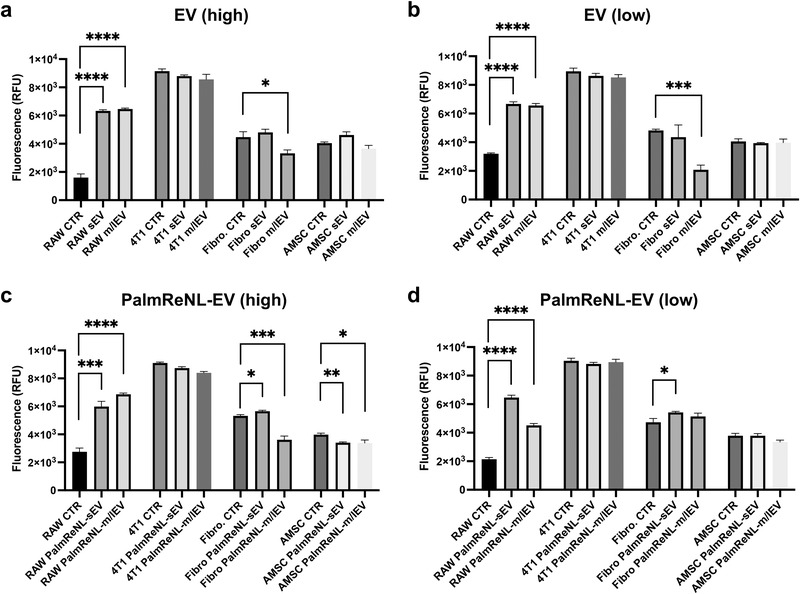
Dramatic increase of the proliferation rate in macrophages after treatment with 4T1 cell‐derived EVs in vitro. a–d) Proliferation of various cell types (RAW 264.7 cells, 4T1 cells, lung fibroblasts, and AMSCs) when cultured for 48 h with or without unmodified EVs and PalmReNL‐EVs (high: 3.0 × 10^9^; low: 3.0 × 10^8^ EVs) Error bars, SD (*n* = 4), *p < 0.05; **p < 0.01; ***p < 0.001; ****p < 0.0001.

To evaluate the physiological significance of cellular EV uptake, we analyzed the proliferation of various cell types (RAW 264.7, 4T1, lung fibroblasts, and AMSCs) when cultured in EV‐depleted media for 48 h with two different concentrations of EVs (high: 3.0 × 10^9^; low: 3.0 × 10^8^ EVs) derived from unmodified 4T1 and PalmReNL‐4T1 cells. Interestingly, both sEVs and m/lEVs at high and low concentrations dramatically increased the proliferation rate in RAW 264.7 cells compared to the other cell types tested (**Figure** **4**a–d). There was no significant difference between unmodified EVs and PalmReNL‐EVs in this effect, indicating that 4T1 cell‐derived EVs, not PalmReNL proteins, mediated this macrophage activation (clearly evident by the change in morphology of cell spreading and formation of dendrite‐like structures) after 48 h of treatment (Figure S4, Supporting Information). Recent studies showed that cells release cytokines not only in free forms but also associated with EVs.^[^ ^29^ ^]^ This cytokine transfer might be related to this macrophage activation by 4T1 cell‐derived EVs, although further studies are needed to test this possible mechanism. Notably, while autologous EVs derived from 4T1 cells did not affect the proliferation of 4T1 cells, both unmodified m/lEVs and PalmReNL‐m/lEVs significantly decreased the proliferation rate in lung fibroblasts (Figure 4a–c), possibly due to the induction of stromal cell differentiation.^[^ ^30^ ^]^

### 2.4 sEVs and m/lEVs Derived from Metastatic Mammary Carcinoma 4T1 Cells Showed Similar Biodistribution and Preferentially Accumulated in the Lung In Vivo

**Figure 5:**
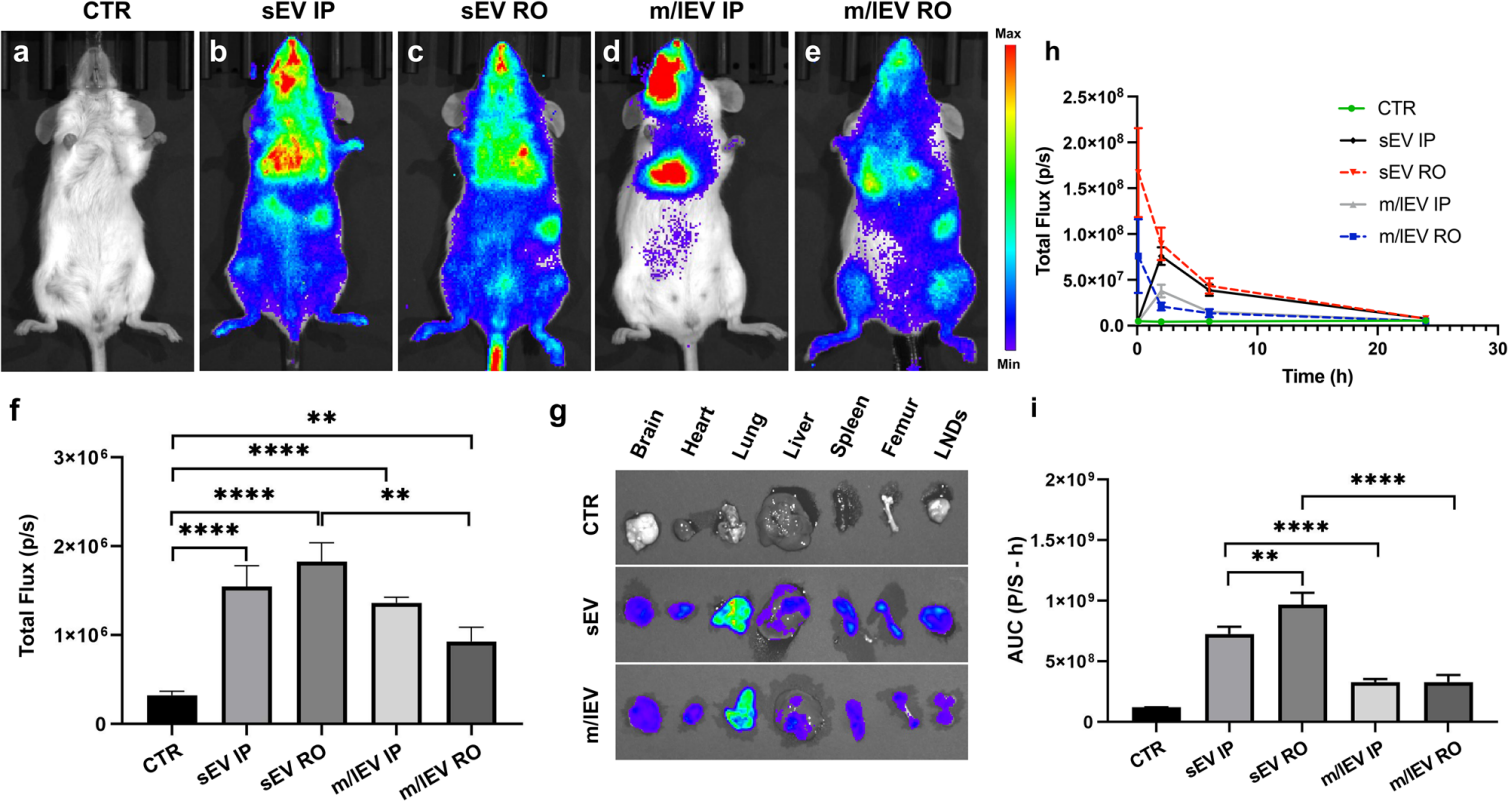
Similar biodistribution and lung tropism of sEVs and m/lEVs derived from metastatic mammary carcinoma 4T1 cells. a–e) 4T1 cell‐derived PalmReNL‐sEVs or ‐m/lEVs (1.0 × 10^9^ EVs/100 µL) were administered retro‐orbitally (RO) or intraperitoneally (IP) in healthy BALB/c mice. Furimazine was RO injected. f) Analysis of bioluminescence signals in (a–e). There was no significant difference between the bioluminescence signals of PalmReNL‐sEVs and ‐m/lEVs IP injected. Error bars, SEM (control [*n* = 10]; sEV IP [*n* = 3]; sEV RO [*n* = 18]; m/lEV IP [*n* = 3]; m/lEV RO [*n* = 15]), **p < 0.01; ****p < 0.0001. g) The ex vivo signals after RO injecting PalmReNL‐EVs, particularly in the lungs, were higher for the reporter EVs when compared to the signal in lungs from control mice. h) The plasma samples were collected from the animals after EV administration. The reporter sEVs displayed significantly higher bioluminescence signals than the reporter m/lEVs in the plasma samples as assessed with furimazine in vitro. The maximum bioluminescence signals were observed at 5 min in the RO injected reporter sEVs and m/lEVs. In contrast, it took 2 h to reach the maximum levels of the bioluminescence signals in the IP injected reporter sEVs and m/lEVs. Error bars, SD (*n* = 3). i) AUC analysis of the bioluminescence signals in the plasma samples over 24 h post‐EV injection. Error bars, SD (*n* = 15), **p < 0.01; ****p < 0.0001.

To determine the biodistribution of 4T1 cell‐derived sEVs and m/lEVs, 1.0 × 10^9^ sEVs or m/lEVs carrying PalmReNL were administered retro‐orbitally (RO) or intraperitoneally (IP) in healthy female BALB/c mice (**Figure** **5**a–e). Both reporter sEVs and m/lEVs distributed throughout the body within five min after RO injections despite the difference of size and membrane composition between these EV classes. The reporter sEVs displayed significantly higher bioluminescence signals (1.8 × 10^6^ ± 8.8 × 10^5^ p s^−1^; *n* = 18; *p* = 0.0016; Figure 5c,f) following RO injections, compared to the reporter m/lEVs RO injected (1 × 10^6^ ± 6.2 × 10^5^ p s^−1^; *n* = 15; Figure 5e,f). The ex vivo signal, particularly in the lungs, was higher for the reporter sEVs RO injected (3.0 × 10^7^ ± 2.4 × 10^7^ p s^−1^; *n* = 3; *p* = 0.02; Figure 5g), when compared to signal in lungs from control mice (3.9 × 10^4^ ± 3.3 × 10^4^ p s^−1^; *n* = 5; Figure 5g). However, there was no significant difference between the lung ex vivo signals for the reporter sEVs and m/lEVs (Figure 5g). Moreover, there was no significant difference between the bioluminescence signals of PalmReNL‐sEVs and ‐m/lEVs IP injected (Figure 5b,d,f).

The bioluminescence signals of the PalmReNL‐sEVs and ‐m/lEVs were also analyzed in plasma samples collected at various time points (5 min, 2, 6, and 24 h) following RO or IP injections (Figure 5h and Figure S5, Supporting Information). The maximum bioluminescence signals were observed at 5 min in the RO injected reporter sEVs and m/lEVs. In contrast, the same reporter EVs took 2 h to reach the maximum levels of the bioluminescence signals following IP injections (Figure 5h). The reporter m/lEVs showed total area under the curve (AUC) for RO: 3.2 × 10^8^ ± 5.7 × 10^7^ p s^−1^ ⋅ h (*n* = 15); and for IP: 3.2 × 10^8^ ± 2.5 × 10^7^ p s^−1^ ⋅ h (*n* = 15) (Figure 5i). The reporter sEVs showed significantly higher bioluminescence signals compared to the reporter m/lEVs (total AUC for RO: 9.6 × 10^8^ ± 9.8 × 10^7^ p s^−1^⋅h [*n* = 15], *p* = 0.0006; for IP: 7.2 × 10^8^ ± 6.0 × 10^7^ p s^−1^⋅h [*n* = 15], *p* = 0.0005, Figure 5i).

To determine if the bioluminescence signals of PalmReNL‐EVs could be enhanced by improved substrate availability in vitro and in vivo,^[^ ^31^ ^]^ we tested a novel Fz analog, fluorofurimazine (FFz), with increased aqueous solubility.^[^ ^31^ ^]^ We found FFz was 1.4‐ and 1.5‐fold more sensitive (sEVs 2.6 × 10^5^ ± 3.9 × 10^3^ p s^−1^, *p* = 0.0012; m/lEVs 1.7 × 10^5^ ± 3 × 10^3^ p s^−1^, *p *< 0.0001) than Fz in vitro (Figure S6a, Supporting Information) with PalmReNL‐sEVs and ‐m/lEVs, respectively. Next, we injected PalmReNL‐m/lEVs via the IP route since IP injected reporter m/lEVs showed consistent total bioluminescence signals in the body and the two injection routes (RO and IP) did not affect the AUC of PalmReNL‐m/lEVs (Figure 5i). Because of its improved solubility, a higher dosage of FFz can be delivered; however, we injected the dosage of 0.25 mg kg^−1^, which is the same as Fz, to compare their sensitivities. PalmReNL‐m/lEVs administered via the IP route exhibited sixfold more bioluminescence when FFz was RO injected as the substrate (2.5 × 10^6^ ± 3.4 × 10^5^ p s^−1^ [*n* = 5], *p* = 0.0005) compared to Fz (4.2 × 10^5^ ± 1.1 × 10^5^ p s^−1^ [*n* = 5], Figure S6b–f, Supporting Information).

### 2.5 Bioluminescence Signals in PalmReNL‐m/lEVs Decreased in Mammary Tumor‐Bearing Mice

**Figure 6:**
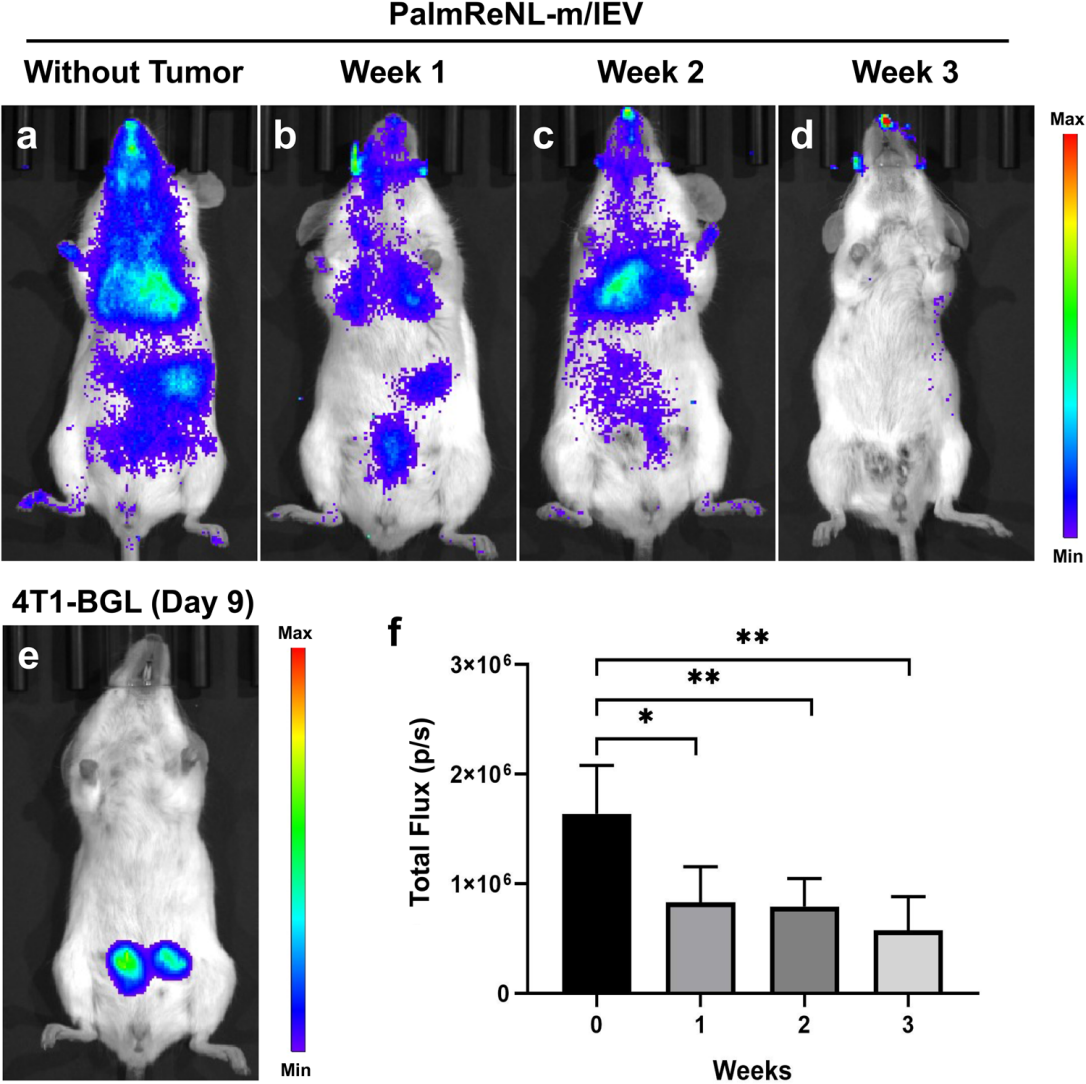
Decreased bioluminescence signals of PalmReNL‐m/lEVs detected after mammary tumor formation. Bioluminescence signals of PalmReNL‐m/lEVs IP injected in mice before and after tumor formation. All the mice were assessed by BLI 2 h after PalmReNL‐m/lEV injection. a) Healthy mice were injected with PalmReNL‐m/lEVs and assessed by BLI with furimazine. b–d) PalmReNL‐m/lEVs (1.0 × 10^9^ EVs/100 µL) were injected into mammary tumor‐bearing mice b) one, c) two, and d) three weeks after tumor implantation. e) Mammary 4T1‐BGL tumors assessed by fLuc BLI with d‐luciferin. f) Quantitative analysis of the bioluminescence signals of PalmReNL‐m/lEVs in mice with or without mammary tumors. Error bars, SEM (*n* = 4), *p < 0.05; **p < 0.01.

Despite their distinct sizes and cellular origins, the reporter sEVs and m/lEVs derived from 4T1 cells behaved similarly in vitro and in vivo under the constraints of our experimental approach. However, the PalmReNL‐m/lEVs produced and retained significantly higher fluorescence signals compared to the PalmReNL‐sEVs, in extracellular spaces (Figure 1k) as well as the intracellular environment after being taken up by cells (Figure 2a–l) likely due to their larger size and symmetric membrane labeling. Tumor cell‐derived m/lEVs have been shown to play a key role in cancer progression by transferring oncogenic receptors to neighboring cells in the tumor microenvironment.^[^ ^32^ ^]^ However, how tumor cell‐derived m/lEVs distribute throughout the body and contribute to metastasis formation has not been determined. To start deciphering the roles that m/lEVs play under both physiological and pathological conditions, we monitored the behavior of PalmReNL‐m/lEVs in mice before and after reporter 4T1‐BGL mammary tumor formation. Interestingly, the bioluminescence signals of PalmReNL‐m/lEVs IP injected in tumor‐bearing mice were significantly lower than that of the mice before tumor formation (**Figure** **6**a–d,f). Seven days (week 1) after tumor cell injection into mammary fat pads, the bioluminescence of IP injected PalmReNL‐m/lEVs was 8.3 × 10^5^ ± 1.9 × 10^5^ p s^−1^ [*n* = 3], *p* = 0.023 (Figure 6b,f). Two weeks after tumor cell injection, the bioluminescence in PalmReNL‐m/lEVs was 7.9 × 10^5^ ± 1.0 × 10^5^ p s^−1^ [*n* = 6], *p* = 0.002 (Figure 6c,f). The longer the time after mammary tumor formation, the more significant was the reduction in the bioluminescence signal. This is reflected by the fact that, three weeks after tumor cell injection, the bioluminescent signal was 5.7 × 10^5^ ± 1.8 × 10^5^ p s^−1^ [*n* = 3], *p* = 0.006 (Figure 6d,f), compared to that of healthy mice before tumor formation (1.6 × 10^6^ ± 1.7 × 10^5^ p s^−1^ [*n* = 7]) (Figure 6a). Mammary 4T1‐BGL tumor growth was assessed by fLuc bioluminescence imaging (BLI) when d‐luciferin was IP injected as the substrate (Figure 6e).

### 2.6 Early Induction of Metastasis by Multiple Doses of Tumor Cell‐Derived m/lEVs in Mammary Tumor‐Bearing Mice

The decrease of the bioluminescence signal of the reporter m/lEVs in the presence of tumors combined with the results of other in vivo and in vitro experiments suggest the involvement of EV‐mediated signaling pathways in the modulation of mammary tumor progression at the lungs. Therefore, in the next set of experiments, we investigated whether m/lEVs play any role in the induction of metastatic lesions.

**Figure 7:**
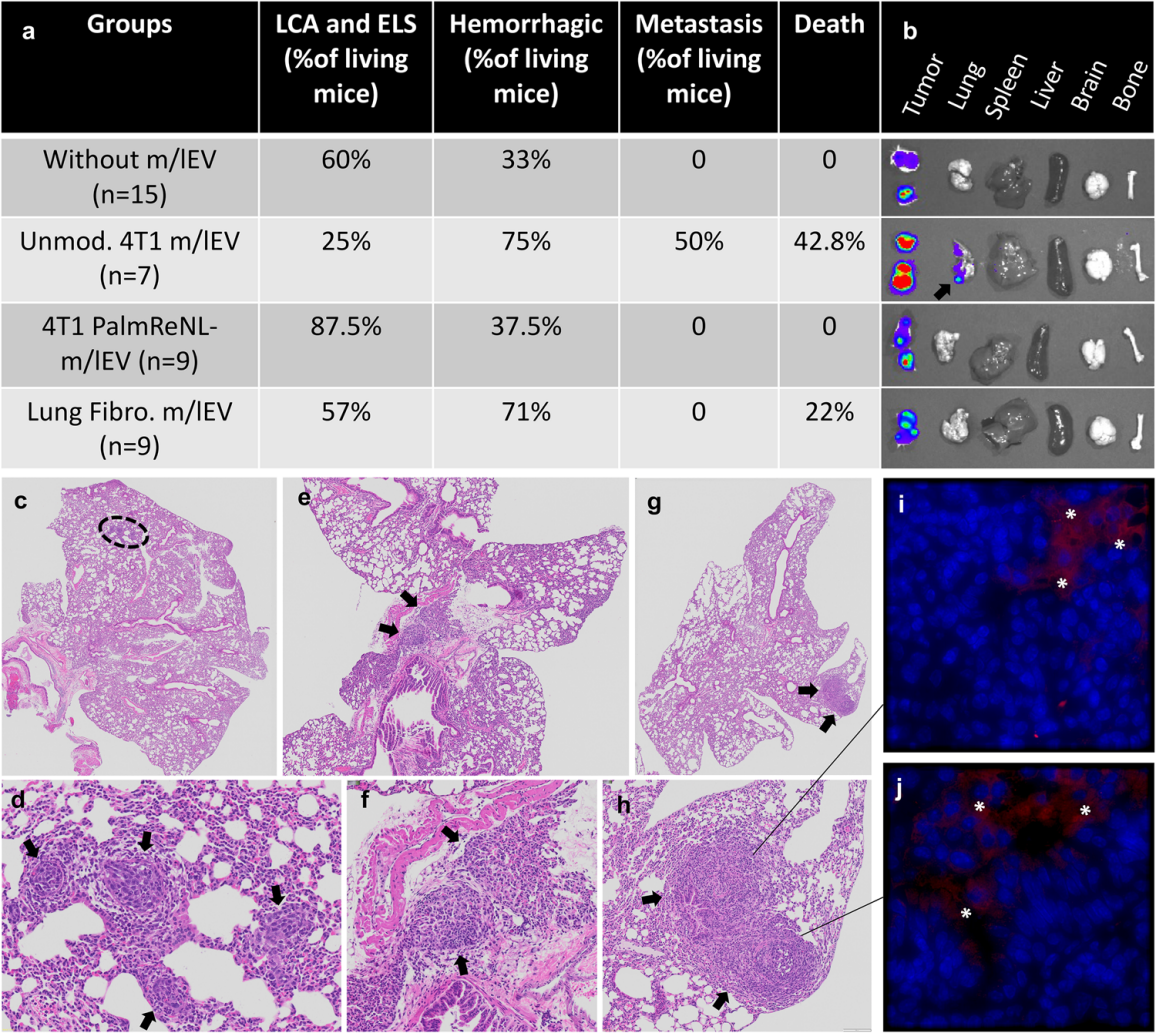
Promotion of early metastasis by multiple doses of tumor cell‐derived m/lEVs in mammary tumor‐bearing mice. a) The table depicts pathological findings including: lymphoid cell aggregates (LCA) or ectopic lymphoid structures (ELS), hemorrhagic lungs, lung metastases, and death. b) In ex vivo BLI data, an arrow points to metastatic foci of 4T1‐BGL cells detected in the lung using d‐luciferin as the substrate. c–h) H&E of mouse lung tissues depicting the metastatic foci from two mice treated with unmodified 4T1 cell‐derived m/lEVs. The images in (c,e,g) are enlarged in (d,f,h). Black arrows indicate metastatic foci. i,j) EGFP‐positive cells (stars) were observed in the lung tissue.

One week after orthotopic implantation of reporter 4T1‐BGL (cells constitutively expressing fLuc and eGFP), 90% of immunocompetent BALB/c mice developed detectable tumors in the mammary fat pad as revealed by BLI (*n* = 40). By two weeks after the implantation of 4T1‐BGL cells, the tumors were still growing steadily. Metastasis was evident in 50% of females that received multiple injections of m/lEVs derived from unmodified 4T1 cells three weeks after the tumor implantation, whereas 4T1‐BGL mammary tumor‐bearing mice without m/lEV treatment did not show any detectable metastasis in the lung (**Figure** **7**a). The metastatic foci were detected ex vivo using d‐luciferin as the substrate (Figure 7b; arrow points to the fLuc bioluminescence signal detecting metastasis in the lung of a mouse treated with unmodified 4T1 cell‐derived m/lEVs).

In addition, the metastatic foci were detected by histological analysis of lung sections following H&E staining (Figure 7c–h). EGFP‐positive cells were observed only in areas near the metastatic foci (Figure 7i,j). Other histological findings included: hemorrhagic lungs were more apparent in mice treated with m/lEVs purified from fibroblasts (71%) and unmodified 4T1 cells (75%) compared to control tumor‐bearing mice without m/lEV treatment; the appearance of ectopic lymphoid structures (ELS) or lymphoid cell aggregates (LCA)^[^ ^33^ ^]^ were predominantly present in lungs of mice treated with m/lEVs isolated from PalmReNL‐4T1 cells (87.5%) (Figure 7a). No other significant pulmonary lesions other than intravascular evidence of systemic inflammation were observed.

**Figure 8:**
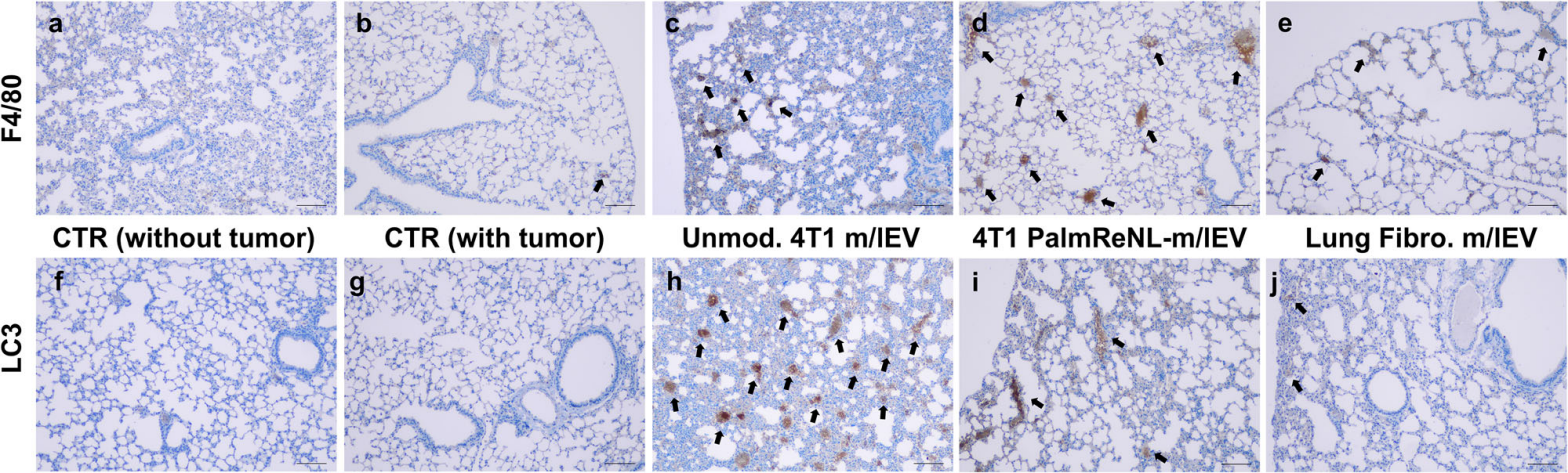
Immunohistochemistry detection of inflammatory foci and the LC3B protein expression. a–e) Tissue sections of lungs from the different experimental groups stained with anti‐F4/80 antibodies. f–j) Tissue sections of lungs stained with anti‐LC3B antibodies. Scale bars, 100 µm. Black arrows indicate F4/80 or LC3 positive regions.

The appearance of ELS and LCA structures within the lung suggested a strong inflammatory response due to the administration of PalmReNL‐m/lEVs. We confirmed this observation through performing immunohistochemistry of lung slides for the localization of F4/80 positive cells to detect foci of inflammation and particularly cells of the mononuclear phagocyte lineage (**Figure** **8**a–e).^[^ ^34^ ^]^ As expected, cells positive for the F4/80 antigen were primarily associated with the lungs of mice treated with m/lEVs derived from PalmReNL‐4T1 cells, localizing primarily to the bronchiolar epithelium and the pulmonary interstitium (Figure 8d). Intriguingly, lungs treated with unmodified 4T1 cell‐derived m/lEVs demonstrated the most aggressive metastatic phenotype, while the F4/80 antigen did not show a remarkable immune response (Figure 8c).

This m/lEV‐mediated promotion of metastasis suggests that 4T1 cell‐derived m/lEVs may modify the tissue microenvironment within the lung in a way that potentiates survival of metastatic cells. Previous reports show that EVs are released during stress and can metabolically reprogram adjacent cells, promoting survival, invasion, and metastasis.^[^ ^35^ ^]^ We hypothesized that m/lEVs may be potentiating the survival of cancer cells within the lung through a similar mechanism. To assess the metabolic health within the lung of m/lEV‐treated mice, we performed immunohistochemistry of the lung slides for the localization of MAP1LC3B (LC3), a key autophagy player often upregulated to promote cancer cell survival during metabolic stress (Figure 8f‐j).^[^ ^36^ ^]^ Remarkably, the expression of the LC3 was upregulated in the lung tissue of mice that developed early metastasis following multiple injections of m/lEVs purified from unmodified 4T1 cells (Figure 8h).

### 2.7 Autophagy Knockout Cell Lines Exhibited Increased EV Release

**Figure 9:**
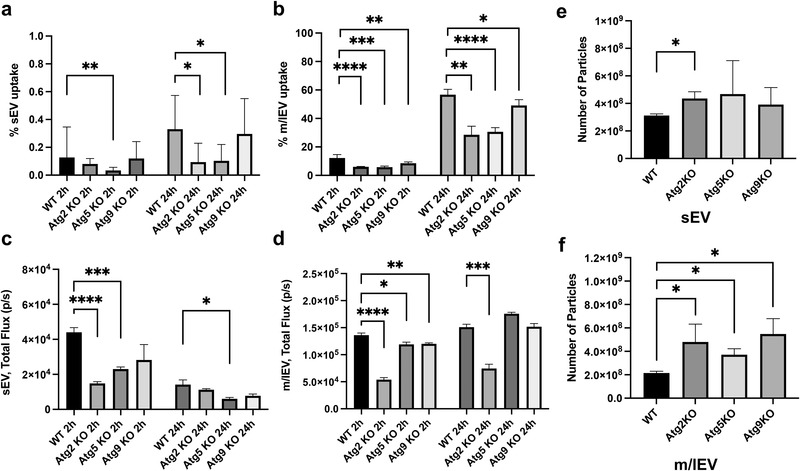
Decreased EV uptake and increased EV release by blocking autophagy in vitro. Uptake of PalmReNL‐sEVs or ‐m/lEVs in control and autophagy knockout (KO) cell lines, analyzed by flow cytometry and measuring bioluminescence. a,b) Flow cytometric analysis of cellular uptake of PalmReNL‐EVs (tdTomato^+^) in U2OS Atg KO cells relative to the parental U2OS cells (WT). The fold change of EV uptake was calculated using tdTomato fluorescence signals in KO cells compared to WT. Error bars, SD (*n* = 3), *p < 0.05; **p < 0.01; ***p < 0.001; ****p < 0.0001. c,d) Uptake of PalmReNL‐EVs determined by measuring bioluminescence signals. Error bars, SD (*n* = 4), *p < 0.05; **p < 0.01; ***p < 0.001; ****p < 0.0001. e) The release of sEVs assessed by NTA increased at the 72 h time point in the Atg2‐KO cell line only. f) The release of m/lEVs increased significantly in all the autophagy KO cell lines compared to WT. Error bars, SD (*n* = 3), *p < 0.05.

Because metabolic stress promotes EV release, we investigated its effect on uptake and release of m/lEV. We tested this in U2OS cells, a well‐characterized autophagy model cell line,^[^ ^37^ ^]^ due to the stark LC3 upregulation seen in our lung metastasis models. Unfortunately, traditional tools used to induce metabolic stress, such as mitochondrial uncouplers or amino acid starvation, result in cell death within 24 h and we were unable to accumulate sufficient m/lEV within the conditioned media at this timepoint. Instead, we characterized the uptake and release of EVs within three different cell lines lacking autophagy‐related genes (Atg2A/B, Atg5, and Atg9A; Figure S7, Supporting Information), which are essential for the induction of LC3‐dependent autophagy.^[^ ^38^ ^]^ Cellular uptake of PalmReNL‐EVs was characterized by assessing tdTomato fluorescence and measuring bioluminescence (**Figure** **9**a–d and Figure S8, Supporting Information). Interestingly, authophagy KO cell lines had opposing effects on the uptake and release of EVs. The uptake of sEVs was reduced mainly in Atg2A/B and Atg5 KO cell lines (Figure 9a). Atg2A/B, Atg5, and Atg9A KO had reduced uptake of m/lEV derived from PalmReNL‐4T1 cells at 24 h in U2OS cells as assessed by flow cytometry (Figure 9b and Figures S8 and S9, Supporting Information). Bioluminescence exhibited reliable results only at 2 h, possibly due to the acid sensitivity of PalmReNL (Figure 9c,d). On the other hand, the number of sEVs isolated from the 2 mL conditioned media at the 72‐h time point increased in the Atg2 KO cell line (Figure 9e), while the number of m/lEVs isolated from the same conditioned media increased significantly in all the autophagy KO cell lines (Figure 9f).

## 3 Discussion

We have developed a novel red‐shifted BRET EV reporter, PalmReNL, for understanding the roles played by the distinct EV classes under physiological and pathological conditions. Here, we visualized sEVs and m/lEVs using PalmReNL in vitro and in vivo. Of note, because of the technical simplicity, the IP administration route of the reporter EVs resulted in more consistent total bioluminescence signals in the body relative to the RO route, hence requiring lower sample numbers to obtain reliable data. In addition to determining the in vivo biodistribution of sEVs and m/lEVs with high sensitivity, PalmReNL allowed us to decipher the possible roles of mammary tumor‐derived m/lEVs in mediating metastasis to the lungs. Although EV research remains restricted by current technical limitations for separating heterogeneous EV populations, the size and EV markers in this study support the successful enrichment of distinct EV classes.

The addition of PalmReNL into the EV membrane did not appear to impair the biological functions of both sEVs and m/lEVs, as demonstrated in vitro with cellular EV uptake using various cell types and in vivo biodistribution studies in mice. However, 4T1 cell‐derived m/lEVs with PalmReNL induced a severe inflammatory response and did not promote early metastasis in the lungs of the mammary tumor‐bearing immunocompetent mice. Our data suggest that host immune activation by antigenic PalmReNL may provide anti‐tumor as well as anti‐metastatic effects.

Fluorescence detection of cellular uptake of PalmReNL‐sEVs gave the false impression of less uptake compared to PalmReNL‐m/lEVs. However, we demonstrated the lack of sufficient sensitivity for detecting fluorescence signals of PalmReNL in the recipient cells when tracking CD63‐mScarlet‐sEVs (Figure 3b, e). Moreover, our analysis of single EVs by flow cytometry revealed that the fluorescence signals in PalmReNL‐sEVs were lower than those of PalmReNL‐m/lEVs (Figure 1k), possibly attributable to their size differences (Figure S1e,f, Supporting Information) and symmetrical labeling of m/lEV membranes (Figure 1d,e). However, an equal number of PalmReNL‐sEVs showed higher bioluminescence signals than PalmReNL‐m/lEVs. This result may indicate that PalmReNL can label more sEVs, while individual sEVs carry probe molecules not detectable by flow cytometry due to the background autofluorescence. Therefore, analysis of bioluminescence signals may more appropriately reflect EV uptake in recipient cells, although the acid sensitivity of PalmReNL limits the long‐term monitoring of cellular EV uptake (Figure 9a,b vs 9c,d).

We concluded that the Palm‐based fluorescent EV probe is not ideal for tracking the fate of sEVs in recipient cells. The rapid diffusion of PalmReNL proteins in the endosomal network likely occurred after cellular uptake. We demonstrated that the reporter sEVs may be processed through the endosomal‐lysosomal pathway by using an acid‐insensitive reporter. PalmGamillus‐sEVs retained the fluorescence signals in the recipient cells compared to the PalmReNL‐sEVs (Figure S3a–d, Supporting Information). Moreover, neutralizing the endosomal pH increased the bioluminescence signal intensity of PalmReNL‐sEVs (Figure 3g). However, these slight signal changes were not detectable by fluorescence microscopy.

Since heterogeneity of EVs was documented,^[^ ^39^ ^]^ it was not surprising that we found different endocytosis mechanisms between sEVs and m/lEVs (Figure 2). EV signaling differs likely because of their various surface receptors and cargo. Additionally, recipient cells may have different activities of macropinocytosis and receptor‐mediated endocytosis.^[^ ^40^ ^]^ Interestingly, unlike other cell types tested, macrophage RAW 264.7 cells took up PalmReNL‐m/lEVs via clathrin‐ and caveolin‐dependent pathways (Figure 2m,q). In addition to phagocytic and macropinocytic EV uptake, this result corroborates findings that tumor cell‐derived EVs are taken up efficiently by macrophages in vivo.^[^ ^41^ ^]^

PalmReNL‐sEVs and ‐m/lEVs produced from 4T1 cells showed similar biodistribution concerning blood circulation and lung tropism in mice, possibly due to the specific proteins expressed on the EV surface.^[^ ^42^ ^]^ Of note, tracking EVs derived from another cell type using the same reporter may result in a different pattern of biodistribution, as previously demonstrated using PalmGRET.^[^ ^6c^ ^]^ The analysis of the blood plasma also demonstrated that both EV fractions decreased quickly, and by 6 h after injection, their blood levels went below half. Interestingly, the circulation time of the reporter m/lEVs became shorter as tumors grew (Figure 6), possibly reflecting the involvement of immune responses^[^ ^41^, ^43^ ^]^ and regulation orchestrated by the tumors and the establishment of premetastatic niches.^[^ ^7^, ^41^, ^42^ ^]^ A study has described how m/lEVs from metastatic melanoma cells enhance lung colonization of less aggressive, non‐metastatic melanoma cells.^[^ ^44^ ^]^ However, further work on m/lEVs has mainly highlighted the ability of m/lEVs to support primary tumor growth and survival.^[^ ^32^, ^35^ ^]^ The early appearance of metastatic foci observed in the present studies following the multiple injections of unmodified 4T1 cell‐derived m/lEVs document potent far‐reaching effects of tumor‐derived m/lEVs and support their future potential as theranostic agents.^[^ ^6^, ^45^ ^]^ Our in vitro assays demonstrated that both sEVs and m/lEVs derived from 4T1 cells trigger strong biological responses in macrophages (Figure 4 and Figure S4, Supporting Information). While 4T1 cell‐derived EVs did not affect the proliferation of lung fibroblasts, AMSCs, and 4T1 cells as strong as macrophages, these cells could be involved in preparing the cell niche that favors metastasis to the lungs during repeated m/lEV injections, a hypothesis that awaits confirmation. Importantly, in the present study, m/lEVs derived from primary fibroblasts did not appear to contribute to the development of metastatic disease in mammary tumor‐bearing mice.

A better understanding of the EV‐mediated systemic cross‐talk between tumor cells and distant cells should aid in developing novel therapeutic approaches. For example, in lung and liver metastasis, exosomes exert their effect through immune cells and stromal cells;^[^ ^41^, ^46^ ^]^ in the bone, they mainly modulate local stromal cells, osteoclasts, and osteoblasts.^[^ ^47^ ^]^ In liver metastasis of pancreatic cancer, macrophages play an essential role in receiving and relaying signals from tumor cell‐derived exosomes.^[^ ^41b^ ^]^ Based on these studies, we hypothesized that m/lEVs modulate metastatic behavior by orchestrating changes in the local tumor microenvironment as well as the systemic activation and recruitment of inflammatory cells at distant metastatic sites. However, results from our experiments demonstrated that the inflammatory cells, likely interstitial macrophages, appear to prevent rather than promote early metastasis (Figure 7). The m/lEV‐mediated potentiation of metastasis is likely multifactorial. This is partially confirmed through the positively stained pockets of LC3 found within the lung tissue, demonstrating the involvement of autophagic signaling within the lung. We hypothesize that tumor‐derived m/lEVs are modifying the tissue microenvironment within the lung through metabolic reprogramming. This alteration can promote proliferation, survival, and immune evasion of metastatic cancer cells in the regions they accumulate. However, further studies are required to characterize whether the upregulation of LC3 is a direct or indirect effect of m/lEV administration. In addition, the influence of autophagy‐related gene knockout on the release and uptake of EVs will need further characterization.

In summary, our new BRET EV reporter system enabled us to track EVs in vitro and in vivo with high sensitivity. By combining non‐invasive in vivo BLI with molecular and cellular analyses, we deciphered the possible role of m/lEVs and LC3‐associated mechanisms in early metastasis (summarized in Figure S10, Supporting Information). Because of the complexity, EV‐mediated signaling likely involves multiple pathways depending on variations of cell types and physiological/pathological conditions. Therefore, further extensive studies are needed to establish commonalities and functional differences of the EV classes. The ability to non‐invasively image cancer‐associated molecular markers will ultimately permit earlier detection and phenotyping of cancer, making possible the development of targeted therapies specific for individual patients.

## 4 Experimental Section

### Plasmid DNA Constructs

All plasmids were constructed using standard PCR cloning protocols. The constructs were sequenced by GENEWIZ (South Plainfield, NJ) before using them for our experiments. For stable reporter gene expression, we constructed a Sleeping Beauty transposon,^[^ ^48^ ^]^ in which the reporter genes were under control of the CAG promoter, by subcloning it into the multiple cloning site of the pKT2/CAGXSP vector^[^ ^19^ ^]^ through recombination cloning (In‐Fusion HD Cloning Kit, Clontech). For the EV reporter, a palmitoylation sequence (MLCCMRRTKQ) of GAP‐43^[^ ^8^, ^49^ ^]^ was genetically fused to the NH_2_ terminus of NanoLuc^[^ ^50^ ^]^ (PalmNLuc), Red‐eNanoLantern (ReNL;^[^ ^16^ ^]^ Addgene plasmid #89 536, gift from Takeharu Nagai), and Gamillus^[^ ^25^ ^]^ for EV membrane anchoring by PCR as reported previously. PalmNanoLuc and PalmReNL were amplified by PCR using (forward) 5′‐tggtggaattctgcagatagccgccaccATGCTGTGCTGTATGAGAAGAACCAAACAGGTCTTCACACTCGAAGATTTCGTTGGGGAC and (reverse) 5′‐cgccactgtgctggatTTACGCCAGAATGCGTTCGCAC, and (forward) 5′‐tggtggaattctgcagatagccgccaccATGCTGTGCTGTATGAGAAGAACCAAACAGGTGAGCAAGGGCGAGGAGGTC and (reverse) 5′‐cgccactgtgctggatTTACGCCAGAATGCGTTCGCAC, respectively. Human CD63 (Addgene plasmid #62 964, gift from Paul Luzio) and mScarlet^[^ ^24^ ^]^ (Addgene plasmid #85 042, gift from Dorus Gadella) were amplified using (forward) 5′‐tggaattctgcagatagccgccaccATGGCGGTGGAAGGAGGAATGAAATG and (reverse) 5′‐ccaccgctacctccacctcctagatctccCATCACCTCGTAGCCACTTCTGATACTCTTC, and (forward) 5′‐tggaggtagcggtggaggtggaagccaggatccgATGGTGAGCAAGGGCGAGGC and (reverse) 5′‐gccactgtgctggatTTACTTGTACAGCTCGTCCATGCCG, followed by combining these amplicons to generate CD63‐mScarlet by overlap extension PCR. The sequence of Gamillus was synthesized as gBlocks (IDT) and a palmitoylation sequence was fused by PCR using (forward) 5′‐tggtggaattctgcagatagccgccaccATGCTGTGCTGTATGAGAAGAACCAAAC and (reverse) 5′‐cgccactgtgctggatTTACTTGTACAGCTCGTCCATGCCG.

### Cell Culture

The labeling efficiency of the palmitoylated reporter was assessed in isolated fractions of sEVs and m/lEVs from 4T1 cells stably expressing PalmReNL‐ or PalmNanoLuc. The murine breast cancer 4T1 cells, murine macrophage RAW 264.7 cells, and primary mouse lung fibroblasts were cultured in DMEM supplemented with GlutaMAX (Gibco), 10% (vol/vol) FBS, and 1% penicillin/streptomycin. Mouse adipose‐derived mesenchymal stromal cells (AMSCs) were cultured in α‐MEM supplemented with 15% FBS and 1% penicillin/streptomycin. Mouse primary lung fibroblasts and AMSCs were isolated as previously described.^[^ ^51^ ^]^ Monoclonal human osteosarcoma cell (U2OS 3XFlag‐HaloTag‐Atg9A, see below) were cultured in RPMI media (Gibco/Thermo Fisher, A4192301) supplemented with 10% FBS and 1% penicillin/streptomycin. All cell cultures were incubated at 37 °C in a 5% CO_2_ atmosphere. Some cultures were treated with either methyl‐*β*‐cyclodextrin (MβCD; 10 mm), chlorpromazine (10 µg mL^−1^),^[^ ^52^ ^]^ concanamycin‐A (0.5 nm), or chloroquine (50 µm). All reagents were purchased from Sigma.

### Generation of the Atg Knockout Cell Lines

A endogenously edited monoclonal human osteosarcoma cell line (U2OS 3XFlag‐HaloTag‐Atg9A) was used as the parental cell line (D.B. and J.S., manuscript in preparation) and subsequent knockout (KO) cell lines were generated by first ligating each genes corresponding sgRNA sequences (Atg2A: CGCTGCCCTTGTACAGATCG, Atg2B: ATGGACTCCGAAAACGGCCA, Atg5: AACTTGTTTCACGCTATATC, Atg9A: aggatatTCGAGAGAAGAAG, FlagTag: atggactacaaagaccatga) into the pX330‐U6‐Chimeric_BB‐CBh‐hSpCas9 backbone vector^[^ ^53^ ^]^ (Addgene plasmid # 42 230; gift from Feng Zhang). These plasmids were co‐transfected with GFP (pMAXGFP, Lonza), and single cells were sorted by FACS into 96‐well plates, then screened and characterized by Western blot. In the case of the Atg9A‐KO cell line, an additional flagTag sgRNA was added when the parental cell line was transfected.

### Cell Proliferation Assays

Cells were seeded in 96‐well plates at a density of 2000 cells per well, and 24 h later treated with sEVs or m/lEVs (3.0 × 10^8^ or 3.0 × 10^9^ EVs) in EV‐depleted media. The viability of the cultures was determined 48 h after EV treatments by using the CellTiter‐Fluor cell viability assay kit from Promega (G6080) following manufacturer's instructions. The filters in the fluorescence plate reader were set for AFC (380–400 nm excitation; and 505 nm emission).

### EV Isolation

EV‐depleted FBS was prepared by 18‐h ultracentrifugation at 100 000 × *g*, 4 °C.^[^ ^54^ ^]^ 4T1, PalmReNL‐4T1, or PalmNanoLuc‐4T1 cells were seeded at 1.5 × 10^6^ cells per 100‐mm cell culture dish. After 24 h the medium was replaced with EV‐depleted medium, and the cells were cultured for an additional 48–72 h. At the end of this culture period, sEV‐ and m/lEV‐enriched fractions were isolated as described previously.^[^ ^8^, ^19^ ^]^ Briefly, conditioned medium was centrifuged at 600 × *g* for 5 min to remove cells and debris. The supernatant was centrifuged again at 2000 × *g* for 20 min at room temperature (RT) to remove apoptotic bodies. More dense m/lEVs were separated from the less dense sEVs by centrifugation (20 000 × *g* for 60 min, 4 °C), using a refrigerated microcentrifuge 5424 R (Eppendorf). Supernatants were filtered through 0.2 µm PES membrane filters (Nalgene, 725–2520) with pressure to remove large vesicles. sEVs were collected by a size‐based EV isolation method with modifications^[^ ^19^, ^55^ ^]^ using 50‐nm membrane filters (Whatman, WHA110603) with holders (EMD Millipore, SX0002500). Briefly, holders with 50‐nm membrane filters were connected to a vacuum manifold (Qiagen) and washed with 5–10 mL of PBS by applying vacuum. Next, the remaining sEV‐enriched fraction in the supernatant was trapped on the membranes, followed by washing with 5 mL PBS. When approximately 200–500 µL of the sample remained, the concentrated sEVs were carefully collected. EV protein concentrations were determined by Bradford assay (Thermo Fisher). All EVs were aliquoted and stored at −80 °C.

### Nanoparticle Tracking Analysis and Zeta Potential Measurement

sEVs and m/lEVs derived from 4T1 cells stably expressing PalmReNL, PalmNanoLuc, or without modification were analyzed using the ZetaView Multiple Parameter Particle Tracking Analyzer (Particle Metrix) following the manufacturer's instructions. EVs were diluted 100‐ to 1000‐fold with PBS or deionized water for the measurement of particle size and concentration. The ZP of EVs was measured after resuspending the EVs in deionized water to a concentration of 8.0 × 10^7^ particles mL^−1^ as previously reported.^[^ ^56^ ^]^

### Flow Cytometric Analysis of Surface Phosphatidylserine in sEVs and m/lEVs, and Cellular Uptake of EVs

For the analysis of EV surface PS, 100 µL of sEV‐ or m/lEV‐fractions derived from 4T1 cells stably expressing PalmReNL were stained with CellTrace Violet (CTV; Thermo Fisher, C34571), followed by removing free dyes with a spin desalting column (BioVision, 6564). CTV‐stained EVs were resuspended to a concentration of 3.5 × 10^9^ particles mL^−1^ in Hanks balanced salt solution (HBSS). 10 × Annexin V binding buffer (0.1 m HEPES pH 7.4, 1.4 m NaCl, 25 mm CaCl_2_) was added to the mixture (final concentration 1×), and incubated with Annexin V‐APC (BioLegend; 8 µg mL^−1^) to a final concentration of 0.7 µg mL^−1^ for 3 h at RT. At the end of the incubation period, the EVs were fixed with 2% paraformaldehyde (PFA, methanol‐free, prepared with 1 × Annexin V binding buffer; final volume 400 µL) for 20 min at RT. Immediately afterwards, the samples were run in the Cytek Aurora spectral cytometer in the MSU Flow Cytometry Core Facility. The signal threshold was set using the CTV fluorescence signal to discern EV signals from the background. Buffer only and buffer with staining reagents were run as assay controls to reliably assess EV signals.

The uptake of PalmReNL‐EVs by the Atg KO cell lines was also analyzed by flow cytometry. Cells were plated in 24‐well plates at a density of 50 000 cells well^−1^, 24 h later the cells were switched to serum‐free media, and 1 × 10^9^ EVs well^−1^ were added. After 24 h, medium was removed and the cells were washed with PBS, trypsinized and harvested. Immediately, the cells were fixed (4% PFA for 20 min at RT) and analyzed by flow cytometry for the fluorescence signal of tdTomato compared to unstained‐cells (negative control) or 4T1 cells stably expressing PalmReNL (positive control).

Flow cytometry data were analyzed with FCS Express v7 (De Novo Software). Gates were drawn based on fluorescence minus one (FMO) controls.

### Fluorescence Microscopy and Bioluminescence Measurements

The uptake of PalmReNL‐EVs by murine macrophages (RAW 264.7), 4T1 cells, primary mouse lung fibroblasts, mouse AMSCs, or U2OS‐Atg‐KO cells was analyzed by fluorescence microscopy and measuring bioluminescence signals. The cells were plated in 96‐ or 24‐well plates at a concentration of respectively 20 000 or 50 000 cells well^−1^. 24 h later the cells were switched to EV‐depleted medium and the reporter 4T1 cell‐derived PalmReNL‐sEVs or ‐m/lEVs were added at a concentration of 3.0 × 10^9^ (for RAW 264.7, 4T1, lung fibroblasts, or AMSCs) or 1–3 × 10^9^ (for Atg‐KO cell lines) EVs well^−1^. At least three wells were analyzed for each treatment group (control, exosomes, MVs). The cultures were allowed to proceed for 24 h. At the end of the incubation period the uptake of the reporter was analyzed by fluorescence microscopy or measuring bioluminescence signals after adding furimazine (Fz; 25 µm) using a VICTOR Nivo Multimode Plate Reader or IVIS Lumina (PerkinElmer).

Phase contrast and fluorescence images of PalmReNL‐4T1 cells, reporter EVs, or cells that were treated with the reporter EVs were taken using All‐in‐one Fluorescence Microscope BZ‐X700 (KEYENCE) or DeltaVision Microscope (GE Healthcare Life Sciences). The cells were stained with 10 µg mL^−1^ Hoechst 33 342 (H3570, Life Technologies) before microscopy was performed. All images were further analyzed using the ImageJ software (imagej.nih.gov).

### Western Blotting

Whole cell lysates and equal numbers of EVs (3.75 × 10^8^ EVs) were derived from unmodified 4T1 and PalmReNL‐4T1 cells and mixed with 4× sample buffer (Bio‐Rad) with *β*‐mercaptoethanol (for detecting TSG101, ALIX, Flotillin‐1, and anti‐RFP) or without *β*‐mercaptoethanol (for detecting CD63). Proteins were separated on a 4–20% Mini‐PROTEAN TGX gel (Bio‐Rad) and transferred to a polyvinylidene difluoride membrane (Millipore, IPFL00010). After blocking with 5% ECL Blocking Agent (GE Healthcare, RPN2125) at RT for 1 h, membranes were probed with primary antibodies overnight at 4 °C at dilutions recommended by the suppliers as follows: anti‐Alix (Proteintech, 12 422), TSG101 (Proteintech, 14497‐1‐AP), flottilin‐1 (BD, 610 820), anti‐RFP (Rockland Immunochemicals, 600‐401‐379), CD63 (Thermo Fisher Scientific, 10628D, Ts63), Atg2A (Cell Signaling, 15 011), Atg2B (Cell Signaling, 25 155), Atg5 (Cell Signaling, 12 994), Atg9 (Cell Signaling, 13 509), followed by incubation with horseradish peroxidase HRP conjugated secondary antibodies at RT for 1 h. The membranes were visualized with ECL select Western Blotting Detection Reagent (GE Healthcare, RPN2235) on ChemiDoc MP Imaging System (Bio‐Rad).

### Dot Blot Analysis

Membrane orientation of PalmReNL in EVs was characterized as reported previously.^[^ ^8a^ ^]^ Both sEVs and m/lEVs carrying PalmReNL (3 × 10^7^ EVs µL^−1^) were 2‐fold serially diluted. Aliquots were incubated in the presence or absence of 1% Triton X‐100 for 30 min at 37 °C. After pre‐wetting a polyvinylidene difluoride membrane (Millipore, IPFL00010) in methanol and equilibrating in transfer buffer, 2 µL of diluted EVs were dotted onto the membrane and blocked in 5% non‐fat dry milk (RPI, M17200‐1000.0) for 1 h at room temperature. The PalmReNL was detected using anti‐RFP (Rockland Immunochemicals, 600‐401‐379) as described under Western blotting methods.

### Proteinase K Protection Assay

sEVs and m/lEVs carrying PalmReNL were split into three identical aliquots (3 × 10^7^ EVs µL^−1^). Proteinase digestion was performed with 1 mg mL^−1^ proteinase K (Qiagen) in the presence or absence of 1% Triton X‐100 for 30 min at 37 °C as reported previously.^[^ ^57^ ^]^ At the end of a digestion period, Fz (25 µm) was added and bioluminescence signals were measured using IVIS Lumina (PerkinElmer).

### Transmission Electron Microscopy

The samples were prepared as previously reported,^[^ ^55^ ^]^ with slight modifications. Isolated EVs (PalmReNL‐sEVs: 6 × 10^7^ EVs/µL, PalmReNL‐m/lEVs: 7 × 10^7^ EVs µL^−1^) were fixed in 1% paraformaldehyde. A formvar‐coated gold grid was kept in a saturated water environment for 24 h and placed on a 50 µL aliquot of EV solution, and allowed to incubate for 20 min while covered. Next, samples were washed and blocked by placing each one face down on top of a 100 µL droplet of the following solutions: PBS (2 ×, 3 min), PBS / 50 mm Glycine (4 ×, 3 min), PBS / 5% BSA (1 ×, 10 min). A 1:100 dilution of anti‐RFP antibody (Rockland Immunochemicals, 600‐401‐379) in 5% BSA/PBS was used for labeling (1 h), followed by six washes in PBS/0.5% BSA. Samples were incubated in a 1:50 dilution of donkey anti‐rabbit immunogold conjugate (Jackson ImmunoResearch, 711‐205‐152) in 5% BSA/PBS (20 min) and washed in PBS (6×) and water (6×). The samples were negative stained with 1% uranyl acetate. Excess uranyl acetate was removed by contacting the grid edge with filter paper and the grid was air‐dried. Samples were observed using a JEOL 1400 Flash Transmission Electron Microscope equipped with an integrated Matataki Flash sCMOS bottom‐mounted camera. The 1400 Flash was operated at 100 kV.

### Bioluminescence pH Titrations

Bioluminescence signals in sEVs and m/lEVs (3 × 10^7^ EVs µL^−1^) carrying PalmReNL were measured at RT using a VICTOR Nivo Microplate Reader (PerkinElmer). EVs were incubated in the presence or absence of 1% Triton X‐100 for 30 min at 37 °C. The solutions consisted of 25 mm pH buffer, 125 mm KCl, 20 mm NaCl, 2 mm CaCl_2_, and 2 mm MgCl_2_ as reported.^[^ ^58^ ^]^ The following buffers were used to adjust pH: pH 4.0–5.0: Acetate Buffer; pH 5.5–6.5: MES Buffer; pH 7.0–8.0: HEPES Buffer.

### In Vivo Tumor and Metastasis Studies

All procedures performed on animals were approved by the Institutional Animal Care and Use Committee of Michigan State University (East Lansing, MI, approval No. PROTO202000273). All mice were purchased from Charles River Laboratory. Eight‐week‐old female BALB/c mice with or without mammary tumors were used for the biodistribution studies of 4T1‐PalmReNL‐EVs (1.0 × 10^9^ particles/100 µL). The mice were imaged with the Fz or fluorofurimazine (FFz^[^ ^31^ ^]^) substrate (5 µg mouse^−1^ in 100 µL PBS). For the induction of tumors, 4T1 cells (2.5 × 10^4^) constitutively expressing BSD‐eGFP and fLuc (BGL) were orthotopically injected into the mammary fat pads of female mice under anesthesia. For the studies analyzing the development of metastasis, following tumor implantation, m/lEVs (3.0 × 10^9^ EVs/100 µL) were injected into mice 3 times per week for 3 weeks (8 treatments in total). 4T1‐BGL tumors were imaged (IVIS Spectrum system, see below) after IP injecting D‐luciferin (3 mg mouse^−1^ in 100 µL PBS). Three weeks after tumor/metastasis induction the mice were imaged with the Fz substrate (5 µg mouse^−1^ in 100 µL PBS), and the following day in vivo and ex vivo fLuc imaging were performed to analyze tumor growth and metastases. Immediately after, mice were sacrificed, dissected, and tissues were fixed (in neutral buffered formalin) and processed for histological analysis following paraffin embedding and H&E staining.

### Lung Immunohistochemistry

Immunohistochemistry for detection of the LC3 protein and the macrophage marker F4/80 was carried out using standard protocols. Briefly, unstained sections of lungs or tumors were deparaffinized and rehydrated, and then incubated in the peroxidase blocking reagent (BioVision cat #K405‐50). Antigen retrieval was performed by boiling the sections in sodium citrate for 20 min. To decrease background staining, the slides were incubated for 1 h in the mouse on mouse blocking reagent (Vector Labs MKB‐2213‐1), followed by overnight incubation with the primary antibodies (LC3B Cell Signaling 3868S rabbit polyclonal, 1:300; or F4/80 Cell Signaling 70076S rabbit monoclonal, 1:200). Next day, the slides were washed and incubated with One‐Step HRP polymer (BioVision cat #K405‐50) for 30 min at RT. The slides were then washed several times and then incubated with the DAB chromogen for 10 min at RT, followed by several washing steps and quick counterstain with Hematoxylin. Slides were then mounted and visualized under the upright microscope (Nikon).

### Bioluminescence Imaging

Bioluminescence analysis of the reporter sEVs and m/lEVs was preceded by the treatment of different concentrations of 4T1‐PalmReNL‐EVs with 25 µm Fz or FFz (Promega). In vitro uptake or in vivo assays for the biodistribution of the reporter sEVs and m/lEVs were imaged with IVIS Lumina or IVIS Spectrum systems (Xenogen product line of PerkinElmer). For in vitro assays, Fz was added to cultures of 4T1 cells, RAW 264.7 cells, lung fibroblasts, AMSCs, or U2OS‐Atg‐KO cells that were treated with the reporter sEVs or m/lEVs prior to BLI. For in vivo imaging, mice were anesthetized with isoflurane using a SAS3 anesthesia system (summit anesthesia support) and an EVAC 4 waste gas evacuation system (universal vaporizer support). Mice were injected retro‐orbitally (RO) or intraperitoneally (IP) with sEVs or m/lEVs (1.0 × 10^9^ EVs/100 µL) with either single or multiple injections (metastasis studies). Five min after RO EV injection or 2 h after IP EV injection, the mice were injected with either d‐luciferin (150 mg kg^−1^; IP), Fz (0.25 mg kg^−1^; RO), or FFz (0.25 mg kg^−1^; RO), and emitted photons were captured with IVIS as described previously.^[^ ^59^ ^]^ Immediately after in vivo imaging, the mice were sacrificed and organs were excised. Organs were washed in PBS, treated with Fz, and imaged with the IVIS system. Bioluminescence signals were analyzed and quantified using the software program Living Image (PerkinElmer).

### Statistical Analyses

All statistical analyses were performed with GraphPad Prism software (GraphPadSoftware). A two‐tailed Student *t‐*test was used for comparison between two groups. One‐way ANOVA followed by Tukey's post hoc test was used for the comparison of three or more groups. Error bars for all the graphs represent mean ± SD or SEM as indicated in each figure. Differences were considered to be statistically significant when the *p*‐value < 0.05.

## Conflict of Interest

The authors declare no conflict of interest.

## Authors Contribution

G.I.P., D.B., and A.A.Z. contributed equally to this work. G.I.P., D.B., A.A.Z., and M.K. conceived and designed the experiments. G.I.P., D.B., A.A.Z., B.D., A.M., V.T., L.K.T., M.P.B., and A.W. executed the experimental work. G.I.P., D.B., A.A.Z., and M.K. carried out the data interpretation and statistical analysis. J.H., J.R.W., T.A.K., M.H.B., and J.S. provided reagents and technical advice. All authors contributed to the writing of the manuscript.

## Peer Review

The peer review history for this article is available in the Supporting Information for this article.

## Supporting information

Supporting Information

## Data Availability

Data sharing is not applicable to this article as no new data were created or analyzed in this study.
