# Supplementary material for: In Vitro and In Vivo Analysis of Extracellular Vesicle‐Mediated Metastasis Using a Bright, Red‐Shifted Bioluminescent Reporter Protein
Source: Adv Genet (Hoboken). 2022 Jan 19;3(1):2100055. doi: 10.1002/ggn2.202100055 (PMC9744575; doi:10.1002/ggn2.202100055)
Supplement: Supplementary file 1 — Supporting Information [file GGN2-3-2100055-s001.pdf]

Supporting Information

In vitro and *in vivo* analysis of extracellular vesicle-mediated metastasis using a bright, red-shifted bioluminescent reporter protein

Authors

Gloria I. Perez*[1,2], David Broadbent*[1,2], Ahmed A. Zarea*[1], Benedikt Dolgikh[1,4], Matthew P. Bernard[1,3], Alicia Withrow[8], Amelia McGill[1], Victoria Toomajian[1,5], Lukose K. Thampy[1,2], Jack Harkema[3], Joel R. Walker[9], Thomas A. Kirkland[9], Michael H. Bachmann[1,6], Jens Schmidt[1,7], and Masamitsu Kanada**[1,3]

Affiliations

[1]Institute for Quantitative Health Science and Engineering (IQ), [2]College of Osteopathic Medicine, [3]Department of Pharmacology & Toxicology, [4]College of Natural Science, [5]Department of Biomedical Engineering, [6]Department of Microbiology & Molecular Genetics, [7]Department of Obstetrics and Gynecology, College of Human Medicine, [8]Center for Advanced Microscopy, Michigan State University, East Lansing, Michigan. [9]Promega Biosciences LLC, 227 Granada Dr, San Luis Obispo, CA.

*These authors contributed equally to this work.

**Correspondence and requests for materials should be addressed to M.K. (email: kanadama@msu.edu).

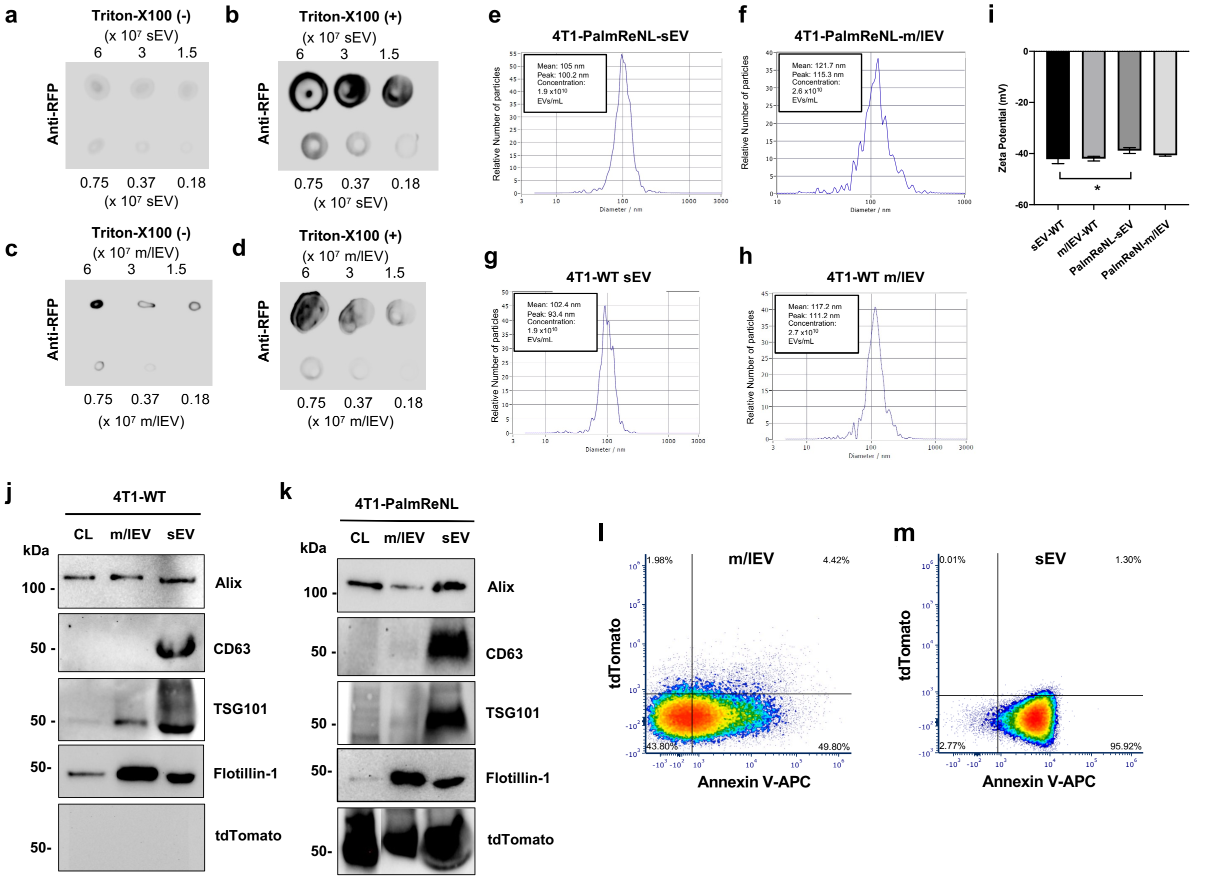

Supplementary Figure 1. Characterization of 4T1 cell-derived PalmReNL-EVs. a-d) Dot blot assays for PalmReNL-sEVs and -m/lEVs with or without Triton-X100 treatment. e-h) 4T1 cell-derived PalmReNL-sEVs and -m/lEVs were analyzed by nanoparticle tracking analysis (NTA). sEVs and m/lEVs derived from unmodified 4T1 cells were analyzed as control. i) Determination of the Zeta potential revealed that the PalmReNL slightly shifted the surface charge of sEVs, but not m/lEVs. Error bars: SD (n = 3); *= $P < 0.05$. j, k) Western blot analysis of exosome marker proteins in m/lEVs and sEVs derived from unmodified 4T1 cells (j) or m/lEVs and sEVs from PalmReNL-4T1 cells (k). l, m) A representative of three independent experiments of Annexin V staining of individual PalmReNL-m/lEVs and -sEVs analyzed by flow cytometry. The tdTomato fluorescence signal represents PalmReNL-EVs. FACS plots were gated for tdTomato^{+} and Annexin V^{+} EVs among the CellTrace Violet (CTV)-stained EVs.

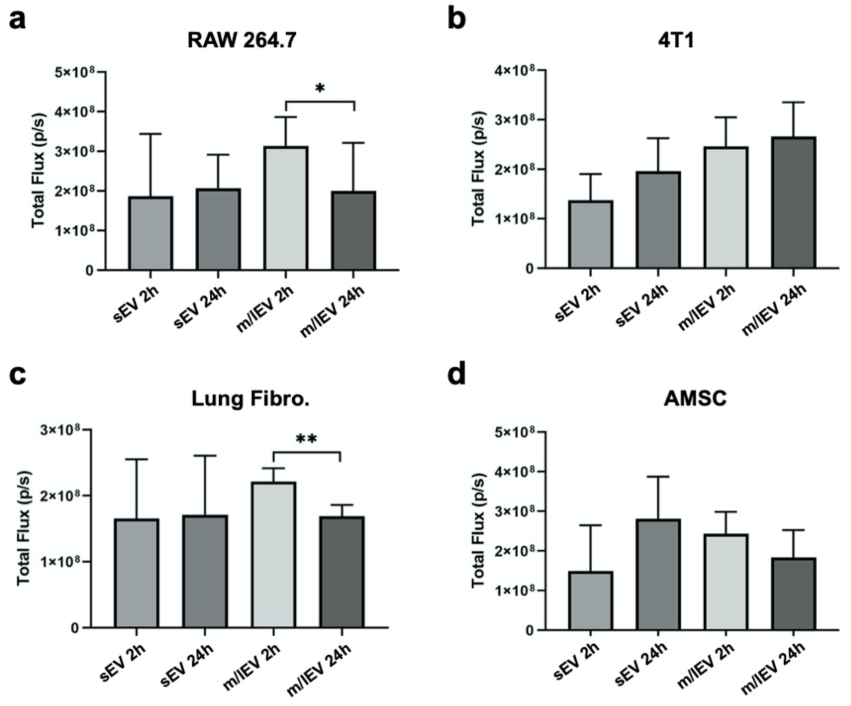

Supplementary Figure 2. Short-term and long-term cellular uptake of 4T1 cell-derived PalmReNL-sEVs and -m/lEVs assessed by measuring bioluminescence. a) Macrophage RAW 264.7 cells. b) 4T1 cells. c) Mouse lung fibroblasts. d) Mouse adipose-derived mesenchymal stromal cells (AMSCs). Error bars: SD (n = 8); *= P < 0.05; **= P < 0.01.

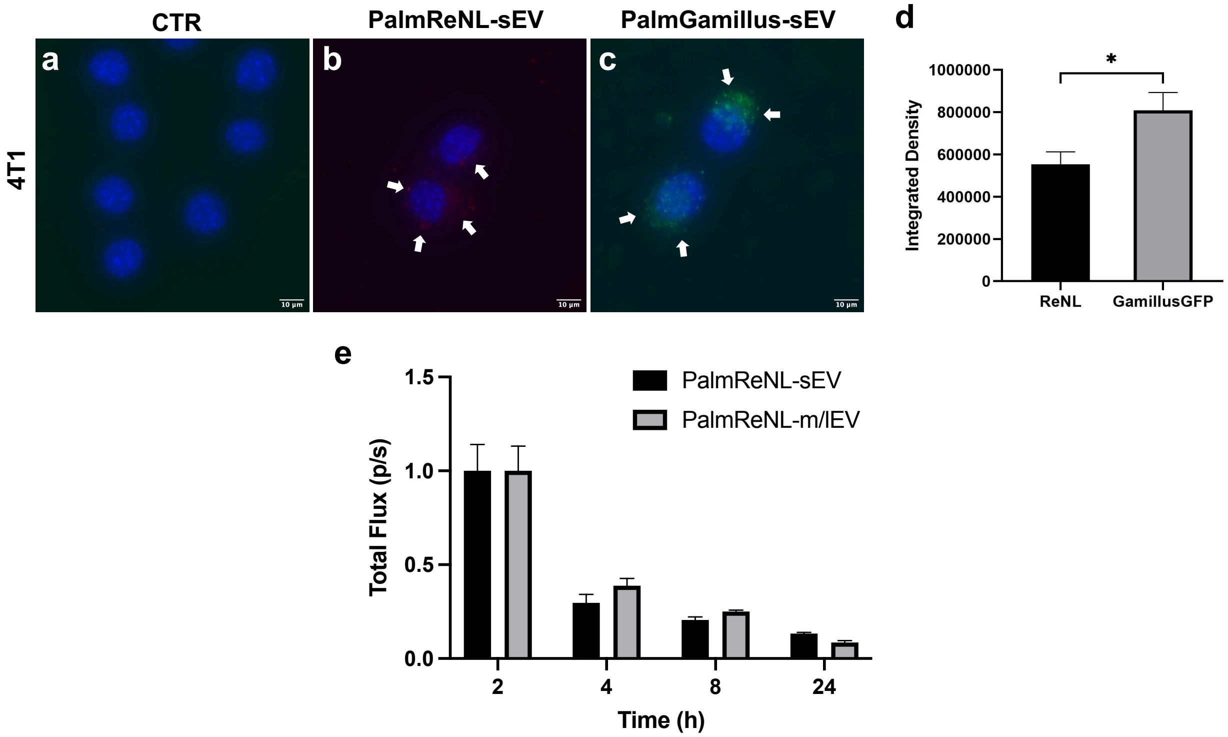

Supplementary Figure 3. Retention of fluorescence and bioluminescence signals in the recipient cells treated with PalmReNL-EVs. a-c) Fluorescence microscopic images of 4T1 cells treated for 24 h with PalmReNL- or PalmGamillus-sEVs. White arrows indicate PalmReNL- or PalmGamillus-sEVs. d) Analysis of the retained fluorescence of PalmGamillus-sEVs in the recipient cells compared to barely detectable fluorescence of PalmReNL-sEVs. e) Bioluminescence signals retained in the recipient 4T1 cells after treating with PalmReNL-sEVs or -m/lEVs for 2 h.

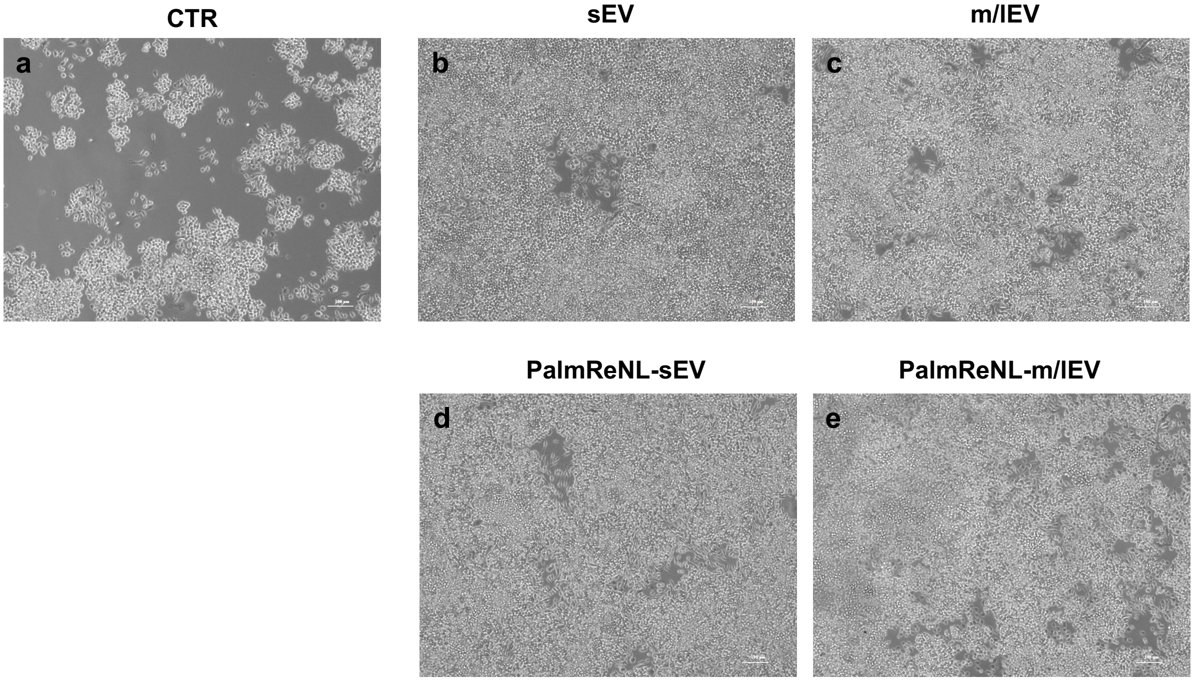

Supplementary Figure 4. a-e) Both sEVs and m/lEVs (3.0×10^9 EVs) activated macrophages regardless of PalmReNL labeling (clearly evident by the rapid proliferation and change in morphology) after 48 h of treatment. Scale bar = 100 μm.

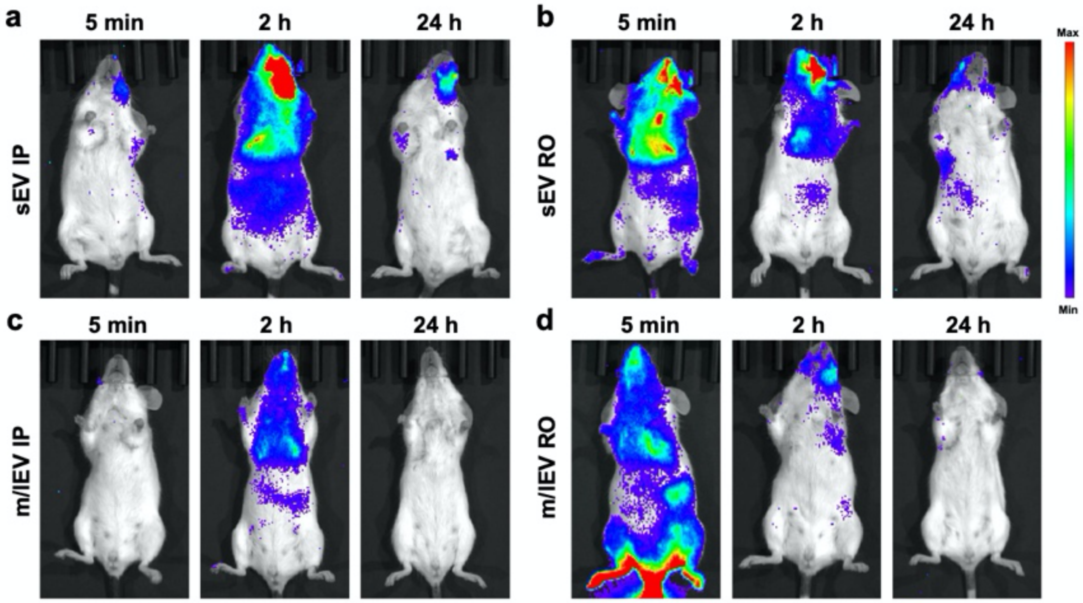

Supplementary Figure 5. Dynamic biodistributions of PalmReNL-sEVs and -m/lEVs following retro-orbital (RO) or intraperitoneal (IP) injections. a-d) Representative biodistributions of PalmReNL-EVs (n = 3). *In vivo* BLI at 5 min, 2 h, and 24 h after injecting PalmReNL-sEVs and -m/lEVs. Furimazine (Fz) was administered via the RO route.

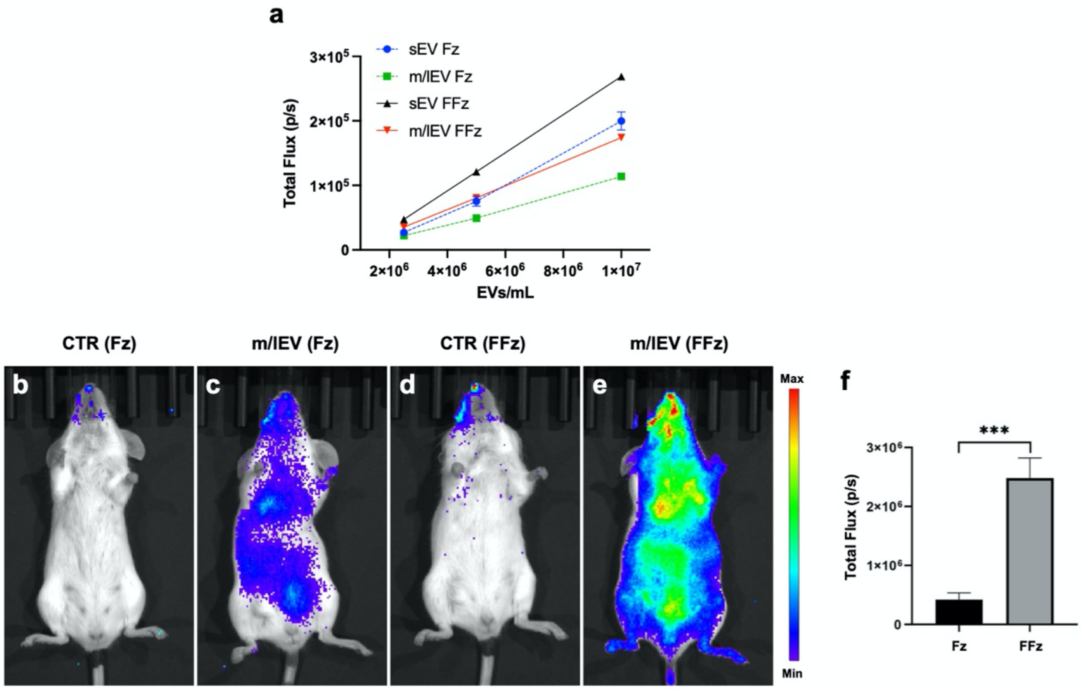

Supplementary Figure 6. Enhanced bioluminescent signals of PalmReNL-m/lEVs when Fluorofurimazine (FFz) was used as the substrate *in vivo*. a) Bioluminescence analysis of PalmReNL-sEVs and -m/lEVs using furimazine (Fz) and fluorofurimazine (FFz) *in vitro*. Error bars: SD (n = 5). b, d) Control mice without PalmReNL-EVs. c, f) The bioluminescent signal of PalmReNL-m/lEVs 2 h post-IP injection using Fz as the substrate. e, f) The sensitivity of the reporter PalmReNL-m/lEVs 2 h post-IP injection was markedly increased when FFz was used as the substrate; Error bars: SEM (n = 5); *** = P < 0.001.

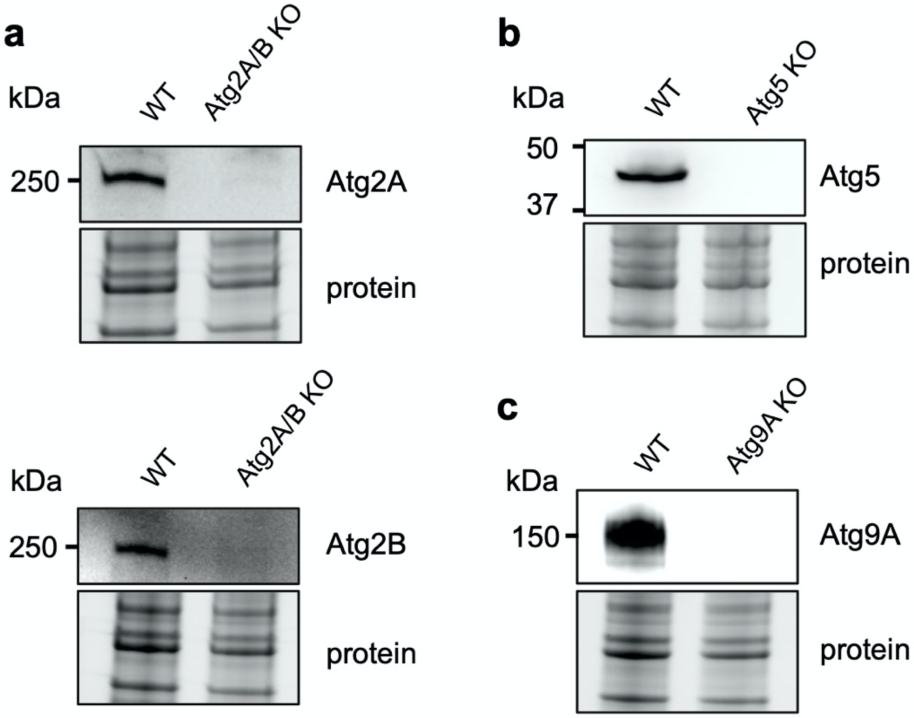

Supplementary Figure 7. Characterization of Atg KO U2OS cells. a-c Western blot characterization of U2OS Atg KO cell lines. a) Atg2A/B KO cells; b) Atg5 KO cells; c) Atg9A KO cells. Whole-cell lysates were shown as a loading control.

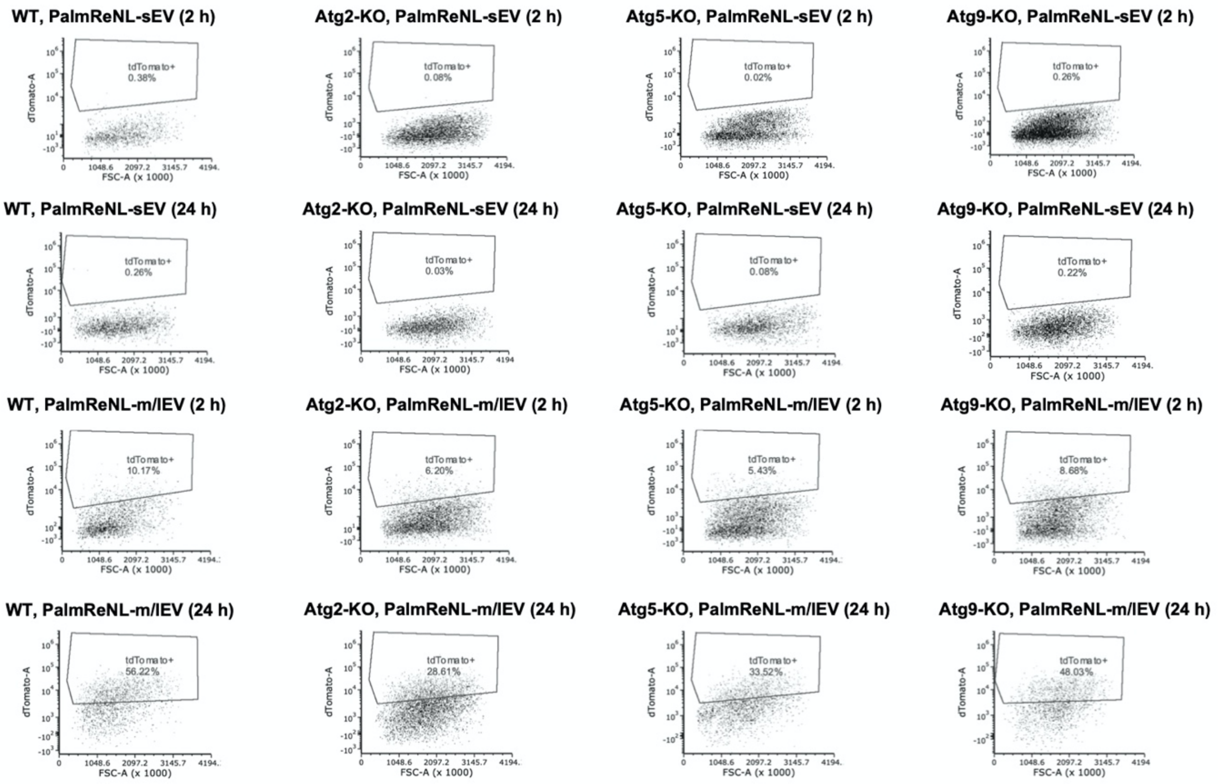

Supplementary Figure 8. Decreased EV uptake of Atg KO U2OS cells. A representative scatter plot of three independent experiments of tdTomato fluorescence signals vs. forward scatter (FSC-A) in Atg KO cells cultured for a short-term (2 h) or a long-term (24 h) with PalmReNL-sEVs or -m/lEVs relative to the parental U2OS cells (WT) in serum-free media. Percent of tdTomato^{+} gated events are shown in each dot plot.

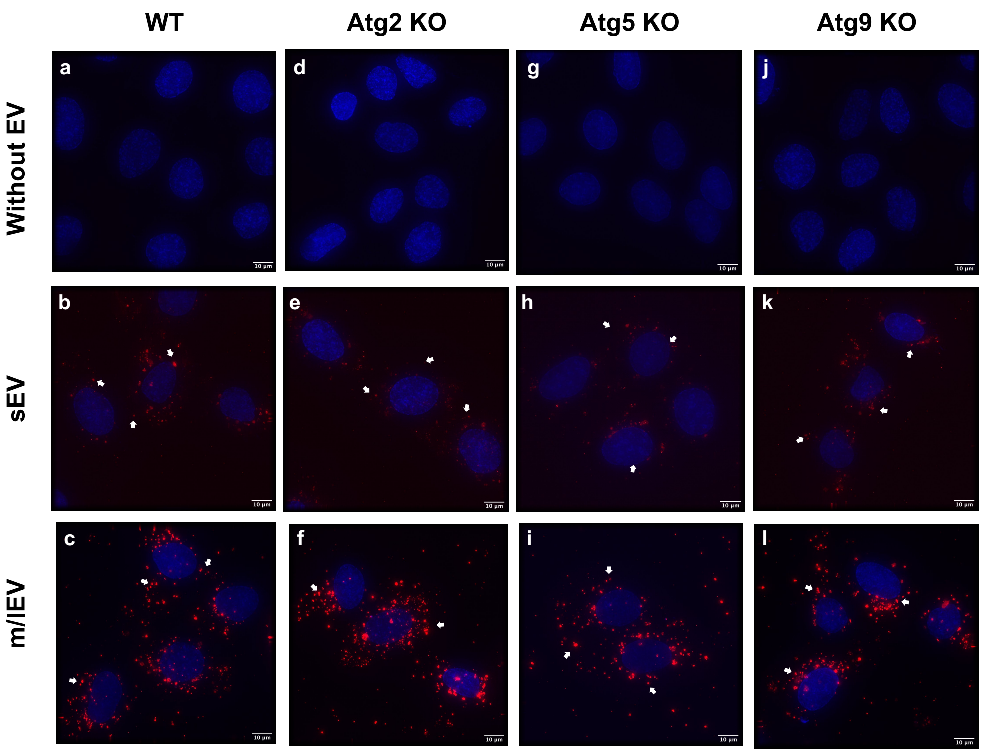

Supplementary Figure 9. Autophagy involvement in cellular uptake of PalmReNL-sEVs and -m/lEVs. a-c) Control WT cells; d-f) Atg2 KO cells; g-i) Atg5 KO cells; j-l) Atg9 KO cells. Punctate signals of RFP (red) were merged with nuclei stained with Hoechst 33342 (blue). Scale bar = 10 µm. Arrows indicate RFP signals in PalmReNL-sEVs and -m/lEVs taken up by the recipient U2OS cells.

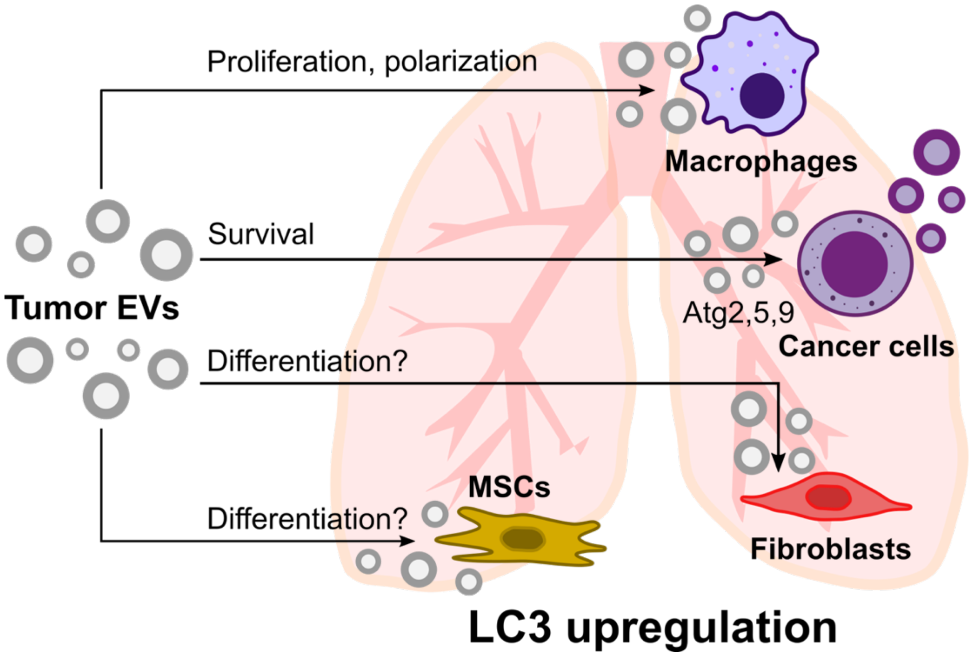

Supplementary Figure 10. Schematic representation of metastatic cancer cell survival in the lung promoted by EVs from primary tumors. EVs released from primary tumors preferentially accumulate in the lung and affect various cell types, including macrophages, cancer cells, fibroblasts, and mesenchymal stromal cells (MSCs). Overall intercellular communication activates LC3 in the lung tissue and suppresses the anti-tumor response. Atg2, 5, and 9 proteins are directly and/or indirectly involved in metastatic cancer cells' EV uptake and release.
